# Organic donor-acceptor heterojunctions for high performance circularly polarized light detection

**DOI:** 10.1038/s41467-022-31186-7

**Published:** 2022-06-15

**Authors:** Danlei Zhu, Wei Jiang, Zetong Ma, Jiajing Feng, Xiuqin Zhan, Cheng Lu, Jie Liu, Jie Liu, Yuanyuan Hu, Dong Wang, Yong Sheng Zhao, Jianpu Wang, Zhaohui Wang, Lang Jiang

**Affiliations:** 1 Beijing National Laboratory for Molecular Sciences, Institute of Chemistry Chinese Academy of Sciences, 100190 Beijing, China; 2 University of the Chinese Academy of Sciences, 100049 Beijing, China; 3 Key Laboratory of Organic Optoelectronics and Molecular Engineering Institution, Department of Chemistry, Tsinghua University, 100084 Beijing, China; 4 Key Laboratory for Micro-Nano Optoelectronic Devices of Ministry of Education, School of Physics and Electronics, Hunan University, 410082 Changsha, China; 5 Key Laboratory of Flexible Electronics (KLOFE) & Institute of Advanced Materials (IAM), Nanjing Tech University, 211816 Nanjing, China

**Keywords:** Electronic devices, Electronic devices

## Abstract

Development of highly efficient and stable lateral organic circularly polarized light photodetector is a fundamental prerequisite for realization of circularly polarized light integrated applications. However, chiral semiconductors with helical structure are usually found with intrinsically low field-effect mobilities, which becomes a bottleneck for high-performance and multi-wavelength circularly polarized light detection. To address this problem, here we demonstrate a novel strategy to fabricate multi-wavelength circularly polarized light photodetector based on the donor-acceptor heterojunction, where efficient exciton separation enables chiral acceptor layer to provide differentiated concentration of holes to the channel of organic field-effect transistors. Benefitting from the low defect density at the semiconductor/dielectric interface, the photodetectors exhibit excellent stability, enabling current roll-off of about 3–4% over 500 cycles. The photocurrent dissymmetry value and responsivity for circularly polarized light photodetector in air are 0.24 and 0.28 A W^−1^, respectively. We further demonstrate circularly polarized light communication based on a real-time circularly polarized light detector by decoding the light signal. As the proof-of-concept, the results hold the promise of large-scale circularly polarized light integrated photonic applications.

## Abstract (web summary)

Here, the authors report a strategy to fabricate multi-wavelength circularly polarized light photodetectors consisting of bilayer donor-acceptor heterojunctions with chiral active layers.

## Introduction

Circularly polarized light (CPL) carrying photons with spin angular momentum has attracted a wide range of interests for its practical applications in circularly polarized ellipsometric tomography, optical communication of spin information, quantum-based optical calculation, biology and medicine, etc.^1–3^. Chiral optoelectronics is a key technology for quantum information and encryption by using the photonic properties of the particular chiral molecules or structures. In order to realize miniaturization and integrated CPL detecting application without using conventional waveplates, there have been increasing demands for optoelectronic devices to detect CPL^4–7^. Hence, organic field-effect transistors (OFETs), as conventional optoelectronic devices^8–14^, provide one of the effective and cutting-edge solutions to convert the direction and intensity of the CPL into sensitive electric signal.

So far, many kinds of chiral materials, including chiral organic materials, chiral perovskite (1D, 2D or quasi-2D), and metal-oxides hetero-chiral structures, have been applied in CPL photodetectors to distinguish the polarized states (Supplementary Tables 1–3)^15–40^. Among them, chiral organic semiconductors combine high optical rotatory power with strong circular dichroism (CD), making them extremely promising as building blocks for CPL photodetector^15–25^. Typical chiral molecular materials in CPL detectors are not only responsible for chiral light absorption but also contribute to charge transport property. The fundamental issue is that the chiroptical response of organic semiconductors requires intrinsic high-level helical structures, which results in low or even no field-effect mobility owing to the asymmetry of the molecular packing. Thus, most of the chiral materials exhibit mobility on the magnitude of 10^−5^–10^−3^ cm^2^ V^−1^ s^−1^ (Supplementary Table 4)^15,16,19,22,23^, which severely restrains the performance of CPL photodetectors such as low photocurrent dissymmetry factor ***g***_**ph**_. Most of the ***g***_**ph**_ of organic CPL detectors based on chiral organic molecules are below 0.1, which hinders their further integration and application. In addition to that, almost all the reported FETs based CPL photodetectors could only work in vacuum or under nitrogen. The instability of organic semiconductors and the existence of semiconductor/dielectric interfacial defects have limited the durability of these devices^41^.

In order to fundamentally address these issues, there are two strategies: (1) the optimization of the chiral materials or structures and (2) the architecture design of the photodetector. Herein, we propose an architecture design strategy by employing the chiral molecules without field-effect electrical behavior and high-mobility organic single crystals to form a bilayer donor-acceptor heterojunction. Specifically, a chiral acceptor molecular layer without FET behavior is inserted between donor semiconductors and source/drain (S/D) electrodes. The geometry is designed following two motivations: (1) The exciton dissociation at the interface of donor-acceptor junction after CP selective absorption. The holes are injected into the p-type semiconductor layer which efficiently contributes to the photocurrent of the device for CPL detection; while electrons are trapped in the chiral active layer which forms a photoinduction electric field to enhance the charge transport property of the semiconductor. (2) The semiconductors/dielectric layer interfacial defects are suppressed without nonconducting chiral acceptor as buffer layer at the interfaces, which reduces the current degradation of the device. Hence, highly stable in-situ CPL photodetectors are achieved based on the heterojunction top-gate bottom-contact (TGBC) photo FET. Especially, by choosing proper donor-acceptor material pairs, multi-wavelength detection can be realized by the structure demonstrated here. The dissymmetry value ***g***_**ph**_ of the CPL photodetectors is up to ±0.24 under 556 nm light illumination in air.

## Results

### Fabrication and properties of CPL photodetector based on heterojunctions

**Fig. 1:**
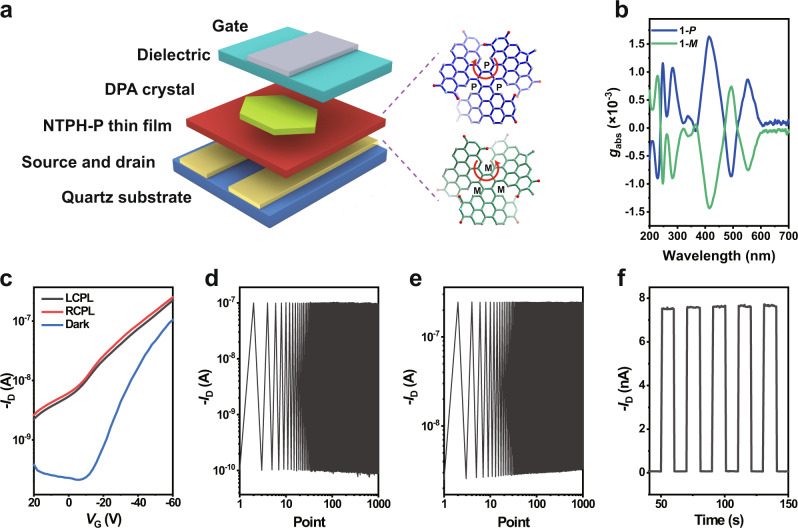
Architecture and device characteristics of the photodetector. **a** Bilayer donor-acceptor heterojunction organic photodetector adopting a device configuration of self-encapsulated bottom-contact top-gate FET in which the chiral active layer and the organic semiconductor layer are NTPH-P thin film and DPA crystal, respectively. The molecular structures of the two chiral forms of the NTPH-P are shown on the right side. **b** ***g***_**abs**_ spectrum of **1-*P*** and **1-*M*** thin films on quartz substrates. **c** Transfer characteristics (recorded at *V*_D_ = −60 V) of photodetector based on **1-*P*** thin film and DPA crystal in dark (blue line) and upon exposure to RCPL (red line) and LCPL (black line) illumination at a wavelength of 556 nm. **d**, **e** Continuous electrical test (recorded at *V*_D_ = −60 V, with *V*_G_ switching from 10 V to −60 V) for the photodetector in dark (**d**) and under 556 nm illumination (**e**). **f** Optical switch characteristics of the photodetector under the illumination at a wavelength of 556 nm with the intensity of 84.88 mW cm^−2^.

For high chiroptical property, we introduce chiral multiple helicene C_2_-symmetric triple [5]helicene based on N-annulated triperylene hexaimide, namely NTPH^42^, as the core chiral active layer in the CPL detector. The pair of enantiomers NTPH-P(P,P,P) (**1-*P***) and NTPH-P(M,M,M) (**1-*M***) (Fig. 1a) exhibits mirror CD response with the dissymmetry factor ***g***_**abs**_ around ±1.6 × 10^−3^ and more than one impressive Cotton effects with multiplet in the visible region (Fig. 1b and Supplementary Figs. 1–3). The ***g***_**abs**_ spectra of the spin-coated films of **1-*P*** and **1-*M*** demonstrate three equal and opposite sharp peaks at 414 nm, 489 nm and 551 nm. Although the enantiomerically pure chiral molecules exhibit fairly good chiral activities in the absorption, the charge transport property of this N-type material based OFET is inefficient (Supplementary Figs. 4a and 5).

In order to realize efficient exciton separation to enhance CPL photocurrent, we employed the bilayer structure of donor-acceptor junction with chiral active layer inserted between high mobility semiconductor and S/D electrodes as shown in Fig. 1a (Supplementary Note 1, Supplementary Figs. 6, 7). 2,6-diphenylanthracen (DPA) was selected as the organic semiconductor layer owing to the high charge carrier mobility, easy preparation process and matching absorption spectrum (Supplementary Notes 2–3 and Supplementary Fig. 4)^43,44^. To ensure sufficient absorption by the chiral active layer (Supplementary Fig. 8), the wavelength of the laser was selected at 556 nm. From the transfer curves of the photodetector based on NTPH-P/DPA crystal shown in Fig. 1c, the mobility, threshold voltage and the on/off ratio of the crystal-based photo FETs are around 1.1 cm^2^ V^−1^ s^−1^, −20 V and 1 × 10^5^, respectively, which are superior to their thin film counterparts (Supplementary Figs. 9–10). Although the introduction of the chiral layer has a slight effect on the contact resistance and properties of the devices, the charge transport properties are still the best in existing reports based on chiral organic system^15,16,19,22,23^ (Supplementary Fig. 11, Supplementary Table 5 and Supplementary Note 4). The crystal-based and film-based detectors exhibit outstanding stability with only 4% (Fig. 1d) and 30% (Supplementary Fig. 12b) on-state current degradation after 500 cycles in dark, respectively, while 130% and 137% (Supplementary Fig. 13b, f) roll-off are observed in the bottom-gate top-contact (BGTC) OFETs based on DPA crystal and film (Supplementary Fig. 14), respectively (Supplementary Table 6, Supplementary Fig. 15 and Supplementary Note 3). Moreover, under the illumination of 556 nm, the on-state current degradation of the single crystal DPA based detector is only 3% (Fig. 1f), further confirming the stability of TGBC DPA crystal-based photo FETs. The impressive stability in air could be attributed to the excellent quality of semiconductor/dielectric interface (Supplementary Fig. 16b), which is the prerequisite of the in-suit CPL detection. The optical switch of **1-*P***/DPA crystal-based photo FET under a 556 nm light illumination is shown in Fig. 1f with the photoresponse up to 10^2^ at intensity of 84.88 mW cm^−2^. The highly stable device performance and obvious photoresponse provide the possibility for further application in real-time information transmission.

**Fig. 2:**
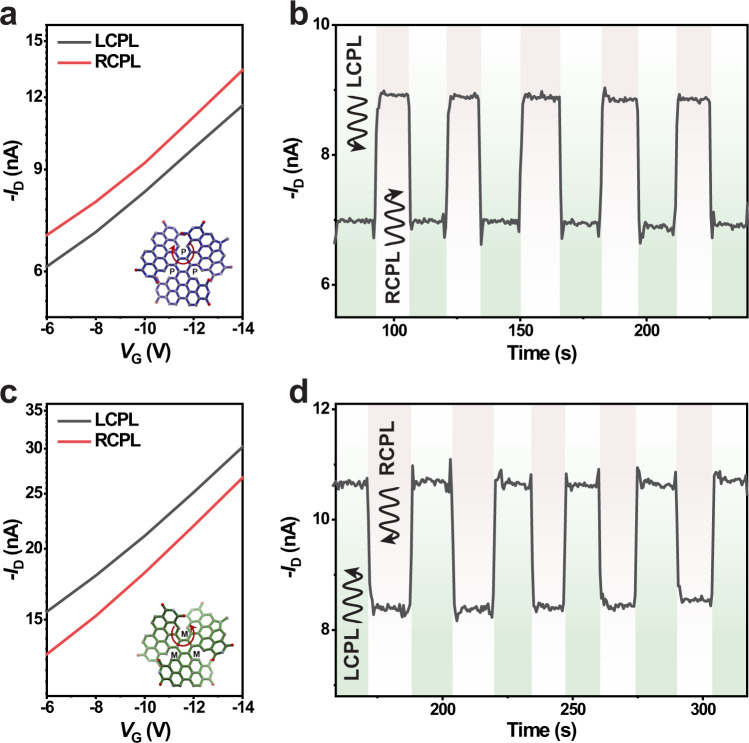
Response of the photodetector to circularly polarized light. **a**, **c** Variation in the transfer characteristics of the photodetector based on **1-*P***/DPA crystal (**a**) and **1-*M***/DPA crystal (**c**) tested upon exposure to RCPL (red line) and LCPL (black line) illumination. **b**, **d** Real-time change in drain current *I*_D_ in response to LCPL and RCPL illumination for the photodetector based on **1-*P*** (**b**) and **1-*M*** (**d**) started with the illumination of LCPL and changed during the test. *I*_D_ was recorded at a constant drain and gate bias of *V*_D_ = −60 V and *V*_G_ = −10 V. The green shading and gray shading are tested with the illumination of LCPL and RCPL, respectively.

**Fig. 3:**
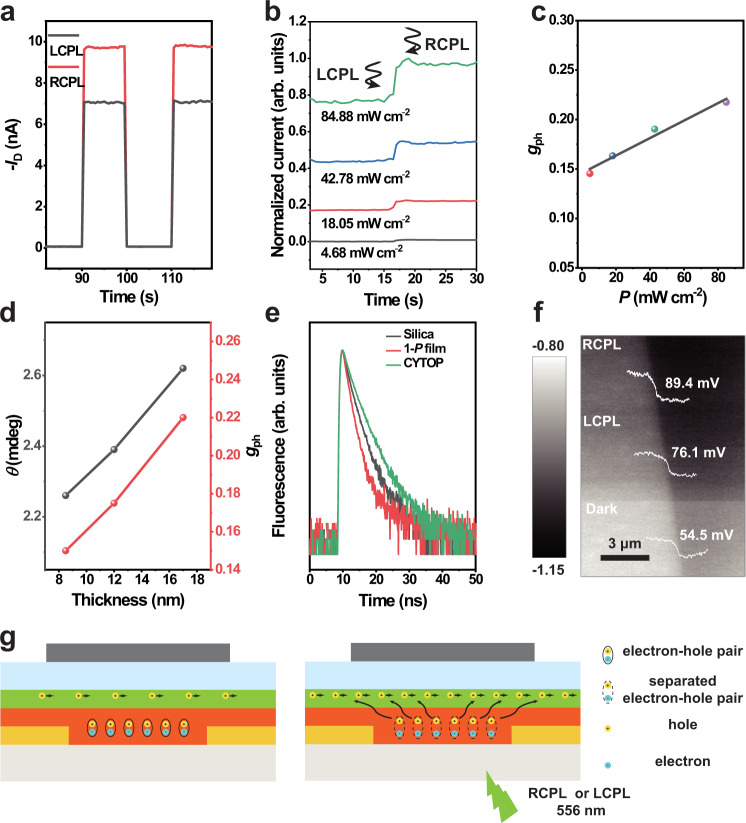
Response and mechanism of the photodetector. **a** Change in drain current *I*_D_ of the detector in response to CPL illumination. The light responses of LCPL and RCPL are superimposed, and the change of the photocurrent can directly correspond to the photocurrent shown in Fig. 2f. **b** Photoresponse of the detector to the switch from LCPL to RCPL illumination under different light intensity. **c** Change of the photocurrent dissymmetry factor exposure to different light intensity illumination. **d** Change of the circularly dichroism signal and the photocurrent dissymmetry factor of the detector based on different thickness of the **1-*P*** film. **e** Fluorescence lifetime of DPA on different substrates, silica, CYTOP and **1-*P*** film. **f** SKPM image of the interface formed by DPA crystal and **1-*P*** film. During the scanning, the bilayer was first placed in the dark condition and then exposed to the laser of 556 nm (from top to the bottom: 556 nm RCPL, 556 nm LCPL, dark). **g** The mechanism of the CPL photo FETs.

The properties of the photo FETs during exposure to left-handed CPL (LCPL) and right-handed CPL (RCPL) illumination are shown in Fig. 2 and Supplementary Figs. 17–19. Photo FETs show distinct photocurrent signal under the illumination of CPL in different polarized directions. From the transfer characteristics (Fig. 2a, Supplementary Fig. 17c), the current increase of **1-*P***/DPA based photo FETs under RCPL irradiation is higher than that under LCPL irradiation. In contrast, opposite phenomenon is observed for the **1-*M***/DPA based photo FETs, where higher photocurrent is obtained under the illumination of LCPL (Fig. 2c, Supplementary Fig. 17d). According to the real-time photocurrent signals, under different polarized light states, from LCPL to RCPL, an obvious change in photocurrent could be obtained (Fig. 2b, d, Supplementary Fig. 18b, d). Moreover, to quantify the degree of response, integral dissymmetry factor ***g***_**ph**_ is used and defined as ***g***_**ph**_ = 2(*I*_LCPL_ − *I*_RCPL_)/(*I*_LCPL_ + *I*_RCPL_), where *I*_LCPL_ and *I*_RCPL_ are the average value of corresponding photocurrent under LCPL and RCPL illumination to ensure the relative accuracy of the values. Here, the ***g***_**ph**_ factor, *R* and EQE values of **1-*P***/DPA crystal-based detector can reach as high as +0.24, 0.28 A W^−1^ and 60%, respectively (Supplementary Table 7 and Supplementary Fig. 20). Meanwhile, the response speed that is characterized by the rise and decay time is around 45 ms for rise and 46 ms for decay in our device (Supplementary Fig. 21) when CPL is shed on the device. The linear dynamic range of the CPL photo FET is calculated to be ∼42 dB with incident-light intensity ranging from 0.8 to 101 mW cm^−2^ (Supplementary Fig. 22a), and the 3-dB frequency is estimated to be around 21 Hz from the frequency-dependent photocurrent of the device (Supplementary Fig. 22b). Besides, compared to the DPA film-based detectors, crystal-based detectors exhibit improved long-term stability, and the photocurrent under the same light polarization has no obvious decay during the test owing to the excellent crystallinity of the semiconductor. The reproducible current signals could be detected when CPL is switched between different polarized directions (Fig. 3a).

In order to further explore the constraints of dissymmetry factor ***g***_**ph**_, we adjusted the light intensity and the thickness of chiral active layer. Factor ***g***_**ph**_ can be regulated by the light intensity of the laser as shown in Fig. 3b, c. With the increase of the intensity, the photocurrent goes up by an order of magnitude, and the factor ***g***_**ph**_ is approximately linearly related to light intensity at the chosen intensity range. In addition, the CPL absorption is highly related to the absorbance of the chiral molecule and the layer thickness, as evidenced by the ellipticity spectra of NTPH-P films with different thickness in Supplementary Fig. 23. As expected, positive correlation between chiral material thickness and ***g***_**ph**_ in the test range is detected (Fig. 3d). The results about the effect of light intensity and thickness of chiral active layer on ***g***_**ph**_ factor indicate that the photocurrent is mainly generated from the photo-induced carriers in the chiral active layer.

### Mechanism of the CPL photodetector

To further probe the working mechanism of the detector, the fluorescence lifetime and scanning kelvin probe microscopy (SKPM) measurements are performed to understand the process of charge transfer. The fluorescence lifetime (*τ*) of DPA crystals on different substrates is tested with the excitation wavelength of 405 nm (Fig. 3e). The results show that the *τ* of DPA crystals on chiral molecule film is significantly reduced compared with that of DPA crystals on silica and CYTOP, which indicates that efficient energy transfer occurs in the donor-acceptor heterojunction (Supplementary Table 8). In addition, SKPM measurements show that the potential of the bilayer heterojunction between the chiral film and the DPA crystal changes under 556 nm irradiation (Fig. 3f, Supplementary Fig. 24). An increase of potential drop at the heterojunction of **1-*P*** film/DPA crystal is detected from dark state to 556 nm illumination, with the potential drop increasing from 54.5 mV (dark state) to 76.1 mV (LCPL irradiation) and then to 89.4 mV (RCPL irradiation). The higher potential drop under RCPL illumination compared with that of LCPL can be attributed to the higher absorbance of **1-*P*** to RCPL, which further confirms the energy transfer between the heterojunction (Fig. 3f). Since no obvious photoresponse signal could be obtained from the OFET based on pure chiral materials under 556 nm illumination, it is speculated that the photocurrent may be caused by the free carriers generated from separated excitons at the interface. Under the dark state, the chiral active layer exists only as ultrathin insulating layer which mainly affects the charge injection of the OFETs. Under the 556 nm laser illumination, similar to the single component OFET^41^, excitons are generated in the chiral active layer upon CP selective absorption. The free holes are injected into the DPA layer which increases the carrier concentration in the channel and enhances the photocurrent of the devices. Meanwhile, the electrons are trapped and accumulate in the chiral active layer, which forms a photoinduction electric field and enhances charge transport property of the holes in the DPA layer. (Supplementary Note 5, Fig. 3g).

### Multi-wavelength detection and information transmission

**Fig. 4:**
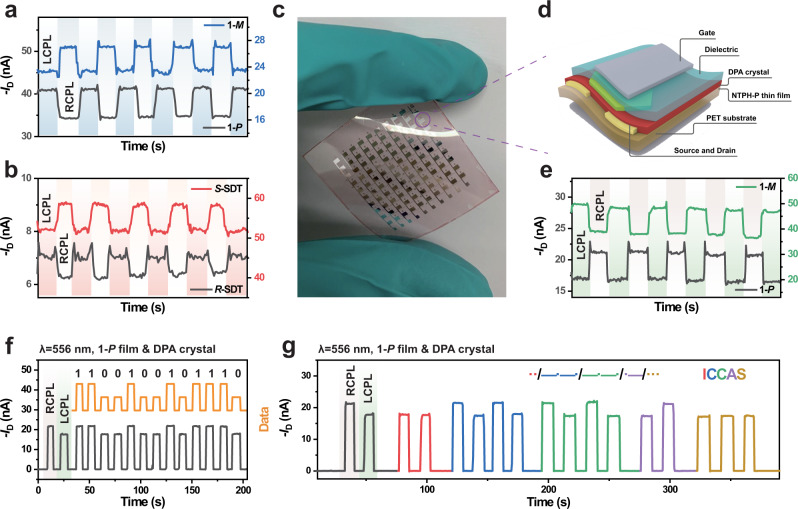
Multi-wavelength circularly polarized light detection. **a** Real-time *I*_D_ change of the detector in response to 488 nm LCPL and RCPL illumination of the NTPH-P/DPA crystal-based photodetector. The blue shading and gray shading are tested with the illumination of LCPL and RCPL, respectively. **b** Real-time *I*_D_ change in response to the 700 nm LCPL and RCPL illumination of the SDT/DPA crystal-based photodetector. The red shading and gray shading are tested with the illumination of LCPL and RCPL, respectively. **c** Optical microscopy image of the flexible device based on **1-*P***/DPA crystal. **d** Architecture of the flexible device fabricated on PET substrates. **e** Real-time *I*_D_ change of the detector in response to 556 nm LCPL and RCPL illumination of the flexible photodetector based on NTPH-P/DPA crystal. The green shading and gray shading are tested with the illumination of LCPL and RCPL, respectively. **f**, **g** Information transmission of flexible photodetector based on **1-*P***/DPA crystal. The green shading and gray shading are tested with the illumination of LCPL and RCPL, respectively.

Since the CPL detection wavelength is dependent on the CPL absorption difference of the chiral molecules^45^, one important advantage of our CPL detectors is they can response to the entire visible light region by selecting proper chiral molecules. For example, we can achieve red (700 nm), green (556 nm) and blue (488 nm) CPL detection based on the donor/acceptor heterojunction strategy. As we mentioned above, chiral molecule NTPH-P exhibits multi CD signals which originate from more than one impressive Cotton effects. Besides 556 nm, 488 nm CPL photodetect can also be realized in NTPH-P/DPA crystal-based detector. The maximum |***g***_**ph**_ | factor is relatively low (around 0.15), which may mainly originate from the lower CP selective absorption (Fig. 4a, Supplementary Figs. 25–26). The detection of 700 nm CPL is realized based on another chiral molecule spiro-fused terylene dimer (SDT), which exhibits a sharp and distinct signal in the ellipticity spectra at 700 nm (Supplementary Fig. 27)^46^. The corresponding relationship between the photocurrent dissymmetry factor and absorption dissymmetry is consistent under the light intensity of 4.58 mW cm^−2^ (Fig. 4b, Supplementary Figs. 28–29).

For real-time in-situ CPL detection, large-area flexible devices are desired for biomedical applications, such as wearable electronics and human skins^47–49^. Primarily, polyethylene terephthalate (PET) is selected as substrate owing to the weak absorption in the test wavelength (Supplementary Fig. 30). Besides, for long term effective detection, the stability of the photocurrent appears to be one of the principal factors. Those devices with continuously increasing or attenuating photocurrent can result in ineffective information transmission (Supplementary Fig. 31, Supplementary Note 6). Among the detectors, we choose the NTPH-P/DPA crystal-based photo FET (556 nm) with the highest photocurrent dissymmetry factor to fabricate flexible CPL detection devices (Fig. 4c, d). The ***g***_**ph**_ factor of device can still reach over ±0.2 (Fig. 4e, Supplementary Fig. 32). Herein, the time-dependent photoresponse of the flexible detector based on **1-*P***/DPA crystal is demonstrated by using the optical on/off switch by converting the polarization of incident CPL randomly for simply simulating the process of quantum information. The photo signals can be converted to information through extraction, definition and transformation by using off state (current in dark) as the data point interval (Fig. 4f). To further confirm that, we decode the light signal based on Morse code encryption^50^. Morse code consists of two basic signals: short dot signals and long dash signals which could be defined by the photocurrent of the device under LCPL and the RCPL. The off state is still an interval of data points, but with a time limit attached. For example, in the signal transmission, the duration of the off states below 10 s represents the interval between dots and dashes, while 10–20 s represents the interval between characters. After simple data processing, we successfully obtain the data of “ICCAS” (Fig. 4g). Through the decryption of data, the content of information transmission can be obtained through electrical signals, which provides the opportunity in optoelectronics for various prospective applications such as quantum information and encryption, biological monitoring, dynamic monitoring and so on.

## Discussion

In summary, a novel CPL photodetector based on chiral semiconductor/high mobility semiconductor heterojunction has been designed and demonstrated successfully, which exhibits high sensitivity, long-term stability and multi-wavelength detectability. Importantly, the device structure demonstrated here averts the high mobility requirement of chiral semiconductors. By optimizing the thickness of the active layer, the dissymmetry factor ***g***_**ph**_ could reach up to over ±0.24, and even for slightly bent flexible devices, this value could still be maintained at a high level of ±0.2. In addition, multi-wavelength detection is realized depending on the multi-peak CD signals of chiral molecules. The device structure provided here could extend the detection wavelength to the whole visible light region, or even to the infrared region, depending on the absorption dissymmetry of chiral molecules.

## Methods

### Materials

The chiral molecules (NTPH-P, SDT) were prepared by spin-coating from 4 mg mL^−1^ chloroform-n-hexane solution on the substrate with S/D electrodes. The different thickness of the thin film could be obtained by different spin-coated conditions at 2000 rpm, 3500 rpm, 5000 rpm for 40 s. The fabrication process of the CPL photodetectors is described in the Supplementary.

### Measurements

UV-Vis spectra were measured with Hitachi (Model U-3010) UV-Vis spectrophotometer on a 1 cm wide quartz plate. Ellipticity spectra were collected on JASCO J-1700 CD spectrometer. The film thickness was determined using Atomic Force Microscope (Cypher ES, Oxford Instruments Asylum Research Inc.). SKPM images were obtained with Bruker Multimode 8. Inverse photoemission spectroscopy measurement was performed using a customized ULVAC-PHI LEIPS instrument with Bremsstrahlung isochromatic mode. Ultraviolet photoelectron spectroscopy was measured with Kratos (AXIS ULTRA DLD). The illumination of liner polarized light was generated through the 556 nm laser (MLG-FN-556), 488 nm laser (MW-BL-488) and 700 nm laser (SC-400, NKT Photonics). The CPL illumination was generated through a quarter-wave plate (Thorlabs, AQWP05M-600). The intensity of LCPL and RCPL light was measured by standard Si detector (Thorlabs, PM100D) to ensure the uniform intensity.

Optoelectronic characteristics were measured by using a semiconductor parameter analyzer Agilent B1500A in the ambient environment at room temperature in dark condition and under illumination. Saturation mobility can be estimated using the equation [Eq. 1]

1 $$\mu =\frac{2L}{W{C}_{i}}\times {\left(\frac{d\sqrt{{I}_{D}}}{d{V}_{G}}\right)}^{2}$$

where *I*_D_ is the drain–source current, *W* is the channel width, *L* is the channel length, *µ* is the field-effect mobility, *C*_i_ is the capacitance per unit area of the gate dielectric layer and *V*_G_ and *V*_T_ are the gate voltage and threshold voltage.

As for the optoelectronic characteristics of the CPL detector, the whole test was explored in ambient conditions. The photoresponse were carried out with different laser with liner polarized light of 488 nm (56.07 mW cm^−2^), 556 nm (84.88 mW cm^−2^) and 700 nm (4.58 mW cm^−2^).

### Contact resistance

The contact resistance of the OFET with the insert chiral layer was calculated by Y-function method.

### Estimation of optoelectronic properties

In order to investigate properties for photo FETs, responsivity (*R*), external quantum efficiency (EQE) and detectivity (D*) were calculated from transfer characteristics coupled with light irradiation. The *R* value is typically defined by the following equations [Eq. 2]:

2 $$R=\frac{{I}_{{{{{{\rm{ph}}}}}}}}{{P}_{{{{{{\rm{in}}}}}}}}=\frac{{I}_{{{{{{\rm{light}}}}}}}-{I}_{{{{{{\rm{dark}}}}}}}}{{P}_{{{{{{\rm{in}}}}}}}}$$

where *I*_ph_ is the photocurrent, *P*_in_ is the incident illumination power on the channel of the device, *I*_light_ is the drain current under illumination, and *I*_dark_ is the drain current in the dark.

The EQE value is typically defined by the following equation [Eq. 3]:

3 $${{{{{\rm{EQE}}}}}}=\frac{R\cdot {hc}}{\lambda q}$$

where *λ* is the incident-light wavelength, *q* is the absolute value of electron charge, *h* is the Planck constant, and *c* is the speed of light.

The D^*^ value is typically defined by the following equation [Eq. 4]:

4 $${D}^{* }=\frac{R}{\sqrt{2q{J}_{{{{{{\rm{dark}}}}}}}}}=\frac{R\sqrt{A}}{\sqrt{2q{I}_{{{{{{\rm{dark}}}}}}}}}$$

where *A* is the channel area.

### Statistics and reproducibility

CPL photodetector can be obtained with similar fabrication for more than 200 independent experiments. More than 100 detectors based on **1-*P*** or **1-*M*** and more than 20 detectors based on ***R*-SDT** or ***S*-SDT** were prepared, and similar results were obtained.

## Supplementary information

Supplementary Information

## Data Availability

The data that support the finding of this study are included within the paper and its Supplementary Information files, or available from the corresponding authors upon reasonable request.

## Code availability

The code is available at: 10.5281/zenodo.6591397^50^.
