# Supplementary material for: Organic donor-acceptor heterojunctions for high performance circularly polarized light detection
Source: Nat Commun. 2022 Jun 15;13:3454. doi: 10.1038/s41467-022-31186-7 (PMC9200767; doi:10.1038/s41467-022-31186-7)
Supplement: Supplementary file 1 — Supplementary Information [file 41467_2022_31186_MOESM1_ESM.pdf]

SUPPLEMENTARY INFORMATION

Organic donor-acceptor heterojunctions for high performance circularly polarized light detection

Danlei Zhu[1,2], Wei Jiang[3], Zetong Ma[1], Jiajing Feng[3], Xiuqin Zhan[1,2], Cheng Lu[1,2], Jie Liu[1], Jie Liu[1], Yuanyuan Hu[4], Dong Wang[1], Yong Sheng Zhao[1], Jianpu Wang[5], Zhaohui Wang[3], Lang Jiang[1,2]

[1]Beijing National Laboratory for Molecular Sciences, Institute of Chemistry Chinese Academy of Sciences, Beijing 100190, China.

[2]University of the Chinese Academy of Sciences, Beijing 100049, China.

[3]Key Laboratory of Organic Optoelectronics and Molecular Engineering Institution, Department of Chemistry, Tsinghua University, Beijing 100084, China.

[4]Key Laboratory for Micro-Nano Optoelectronic Devices of Ministry of Education, School of Physics and Electronics, Hunan University, Changsha 410082, China.

[5]Key Laboratory of Flexible Electronics (KLOFE) & Institute of Advanced Materials (IAM), Nanjing Tech University, Nanjing 211816, China.

Corresponding author email: ljiang@iccas.ac.cn

Supplementary Figures

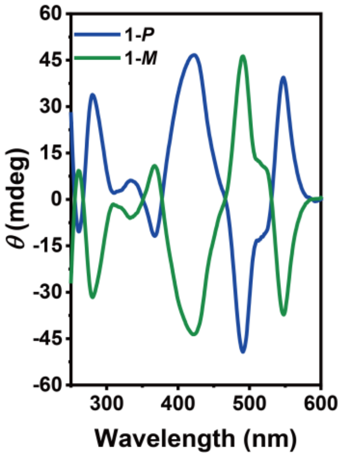

Supplementary Figure 1. Ellipticity spectrum of the chiral materials in solution. Ellipticity spectrum of **1-*P*** (blue line) and **1-*M*** (green line) in chloroform (10^{-5} M).

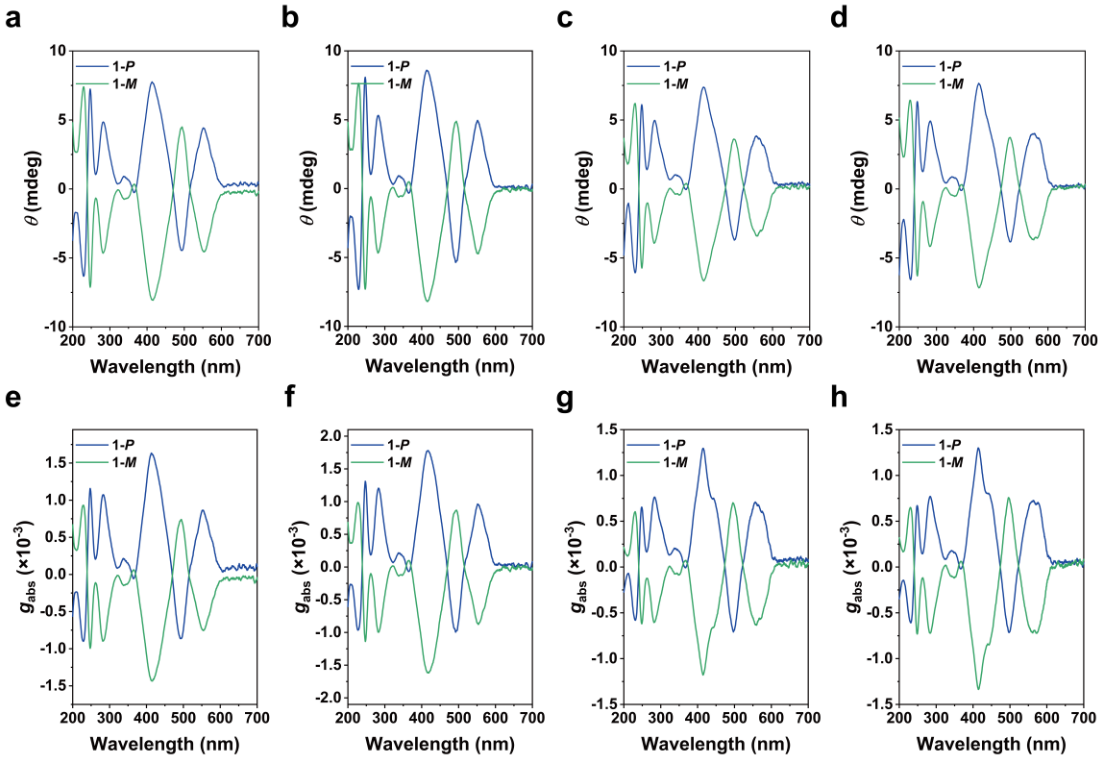

Supplementary Figure 2. Ellipticity and g_{abs} characteristics of the chiral active layers. Ellipticity and g_{abs} spectra of NTPH-P films (**a**, **e**), NTPH-P films prepared with CYTOP (**b**, **f**), NTPH-P films evaporated with DPA film (**c**, **g**) and NTPH-P films prepared with DPA film and CYTOP (**d**, **h**).

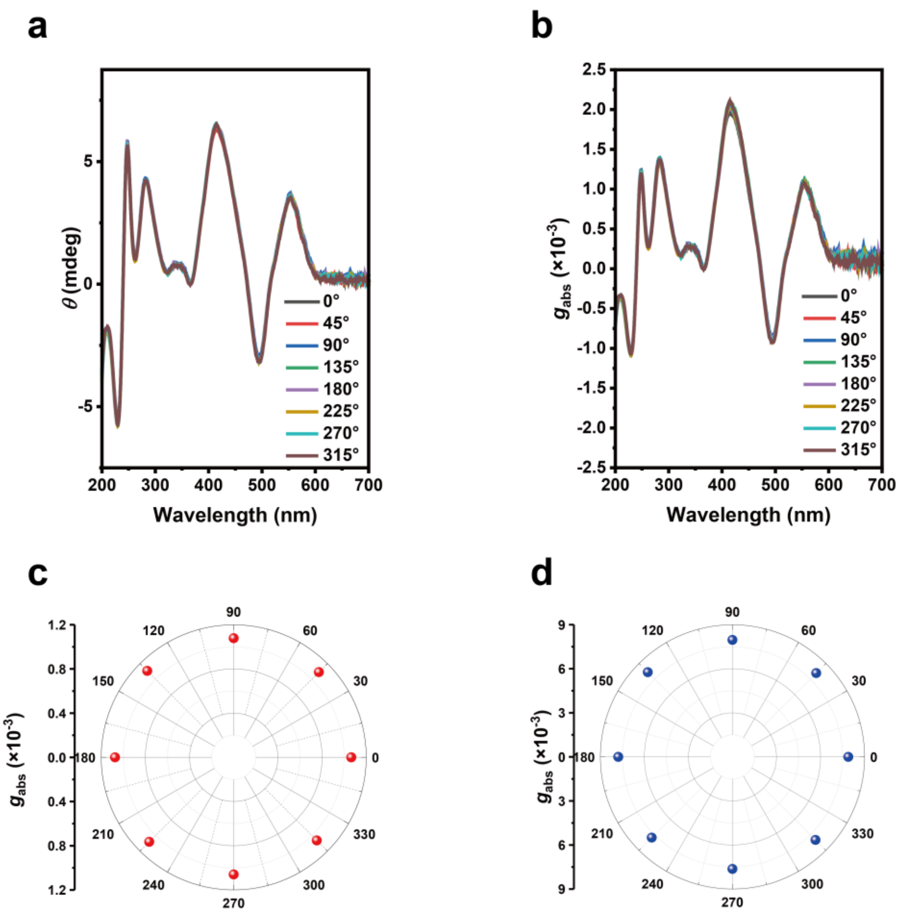

Supplementary Figure 3. Ellipticity and $\boldsymbol{g_{abs}}$ characteristics of 1-*P* thin film. Ellipticity and $\boldsymbol{g_{abs}}$ spectra of the **1-*P*** thin film with different azimuthal rotations (**a**, **b**). Evolution of the $\boldsymbol{g_{abs}}$ of the film with angle at 556 nm (**c**) and 488 nm (**d**), respectively.

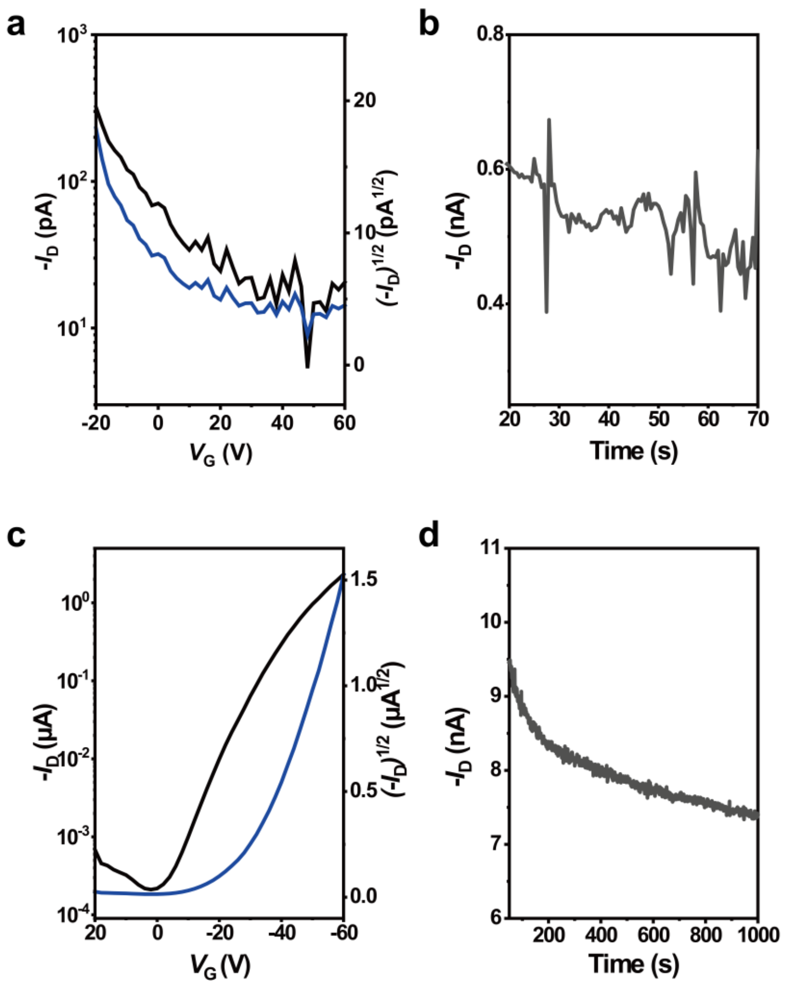

Supplementary Figure 4. The OFET characteristics of the single component. Transfer characteristics of the TGBC OFETs based on neat **1-*P*** film (**a**) and DPA crystal (**c**). Change in drain current I_D of the OFETs based on only **1-*P*** film (**b**) and DPA crystal (**d**) in response to the LCPL and RCPL illumination. The current does not change significantly under different state of CPL illumination.

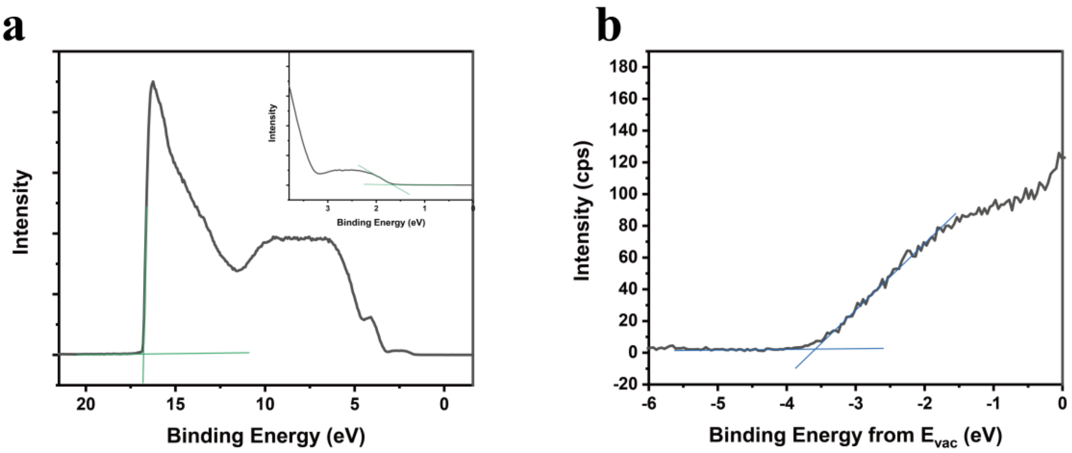

Supplementary Figure 5. Energy levels of NTPH-P thin film. HOMO (a) and LUMO (b) energy levels of NTPH-P thin film.

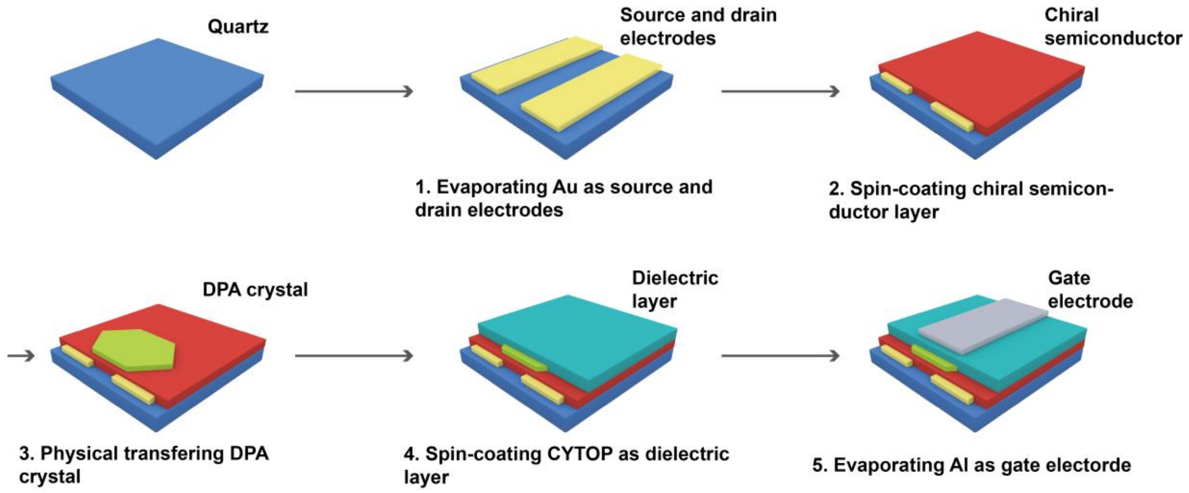

Supplementary Figure 6. The fabrication process of the photodetector. A schematic for the fabrication of the photodetector based on chiral semiconductor film/DPA crystal.

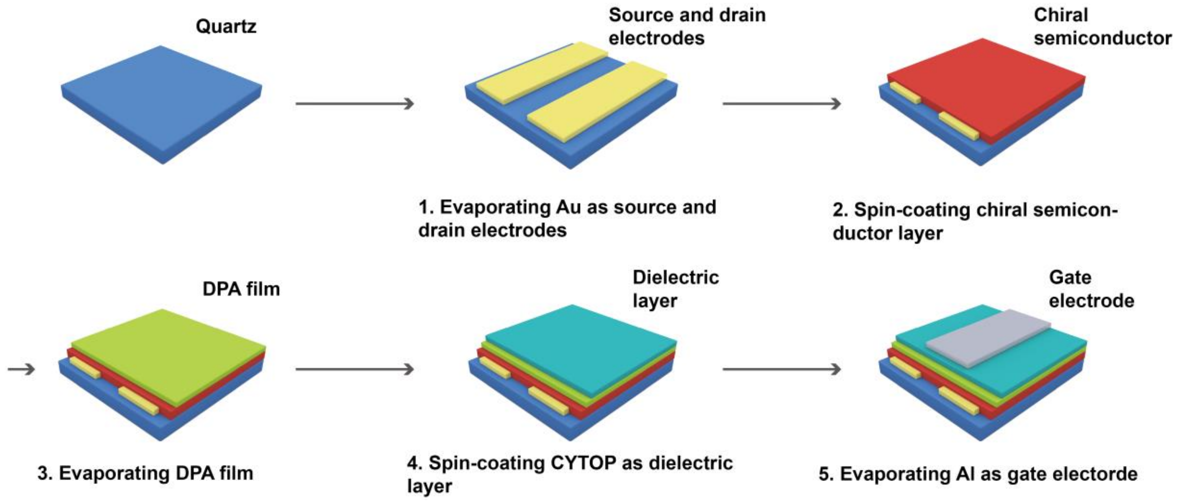

Supplementary Figure 7. The fabrication process of the photodetector. A schematic for the fabrication of the photodetector based on chiral semiconductor film/DPA film.

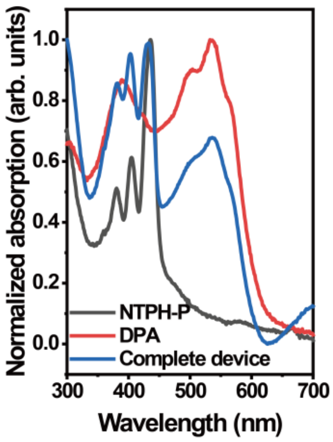

Supplementary Figure 8. Normalized absorption spectrum of the thin films. Normalized absorption spectrum of NTPH-P thin film, evaporated DPA thin film and the NTPH-P/DPA film complete device.

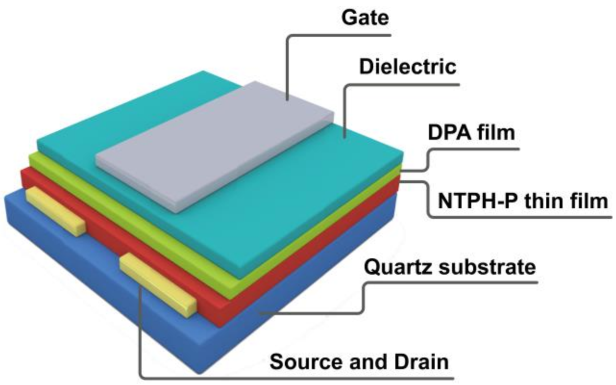

Supplementary Figure 9. Architecture for the device. Architecture for photodetector based on NTPH-P thin film and DPA film.

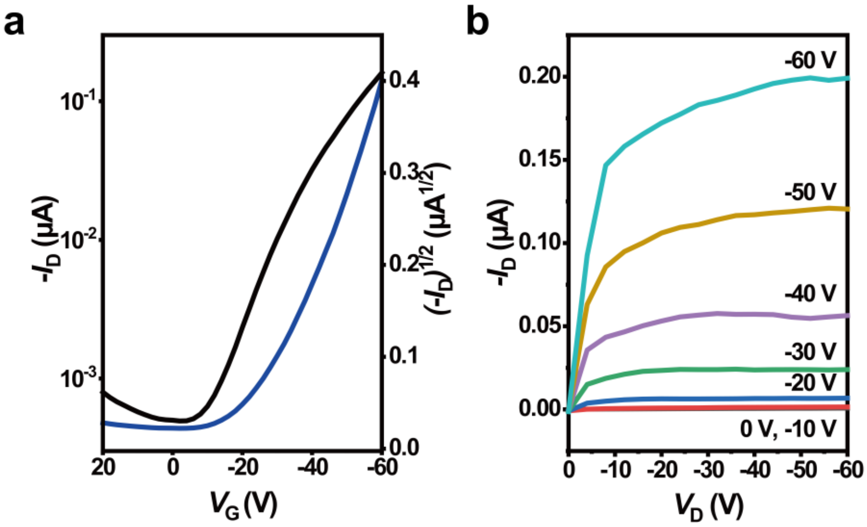

Supplementary Figure 10. The OFET characteristics of the photodetector. Transfer characteristics (recorded at V_D = -60 V) (**a**) and output characteristics (**b**) of OFET based on **1-*P*** thin film and DPA thin film.

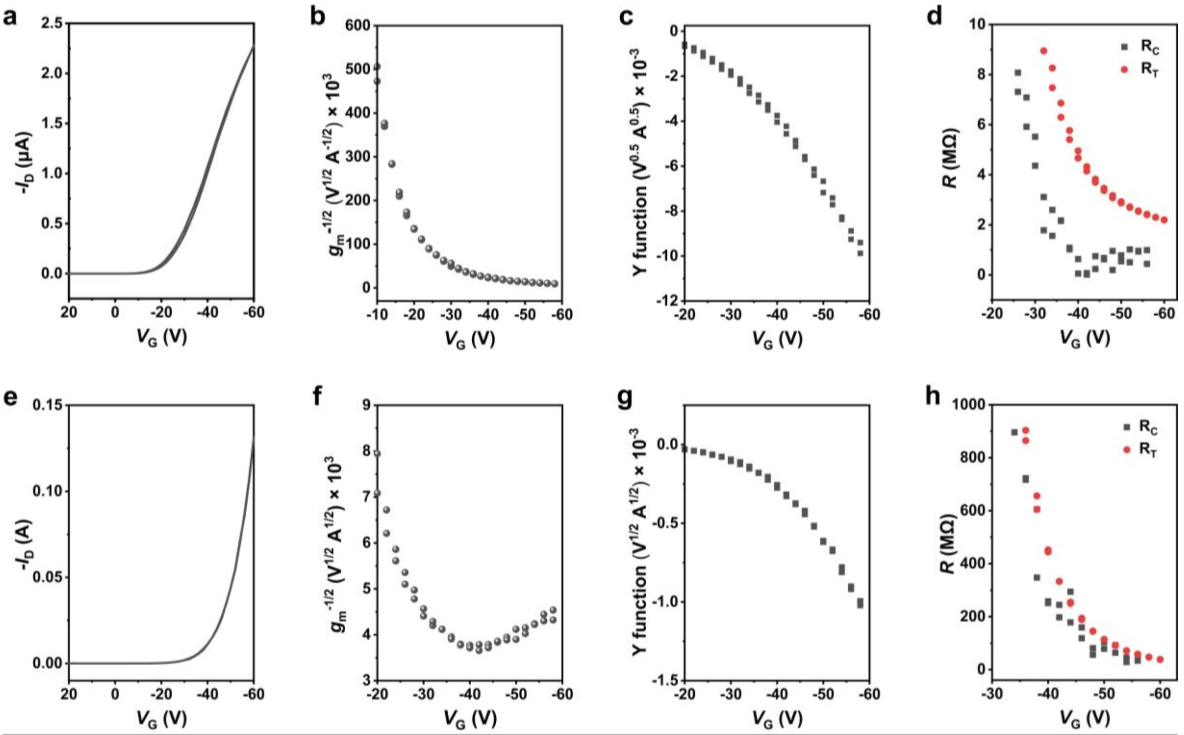

Supplementary Figure 11. Contact characteristics of the FETs. Transfer characteristics in linear regime (recorded at V_D = -5 V) (**a, e**), g_m-V_G characteristics (**b, f**), Y function-V_G characteristics (**c, g**), and resistance data (**d, h**) for FET (**a-d** are the characteristics based on DPA crystal TGBC FET, **e-h** are the characteristics based on **1-*P***/DPA crystal TGBC FET).

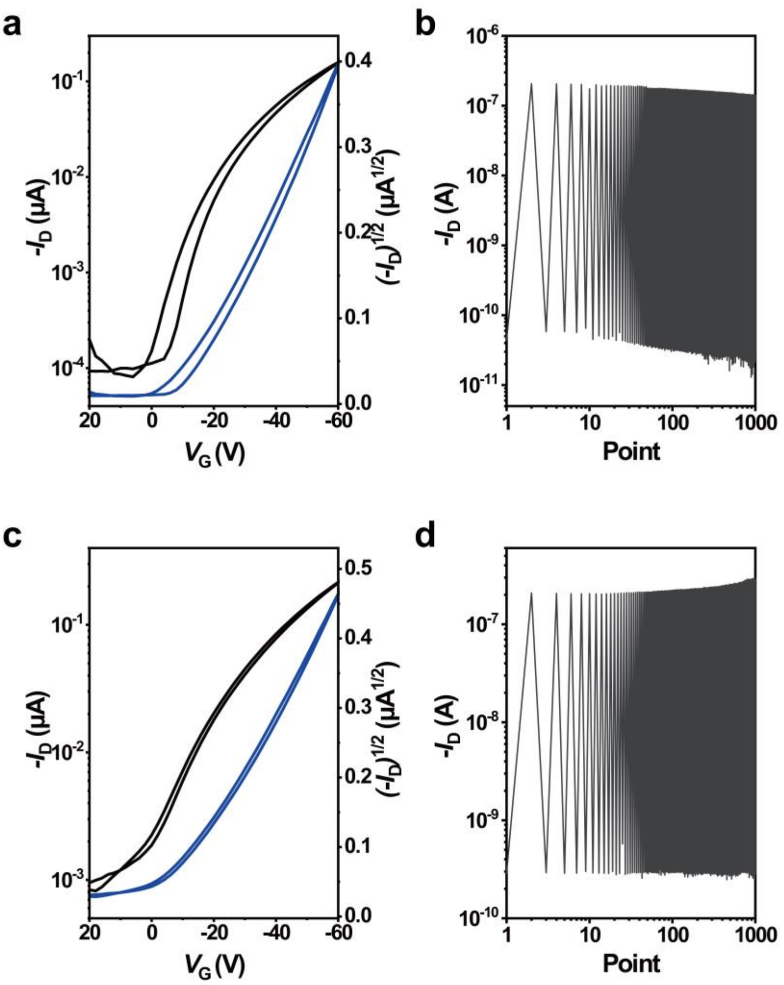

Supplementary Figure 12. The OFET characteristics of the photodetectors. Dual sweep transfer characteristic and continuous electrical test (recorded at V_D = -60 V, with V_G switching from 10 V to −60 V) for TGBC OFETs based on **1-*P***/DPA film at dark state (**a, b**) and under 556 nm illumination (**c, d**).

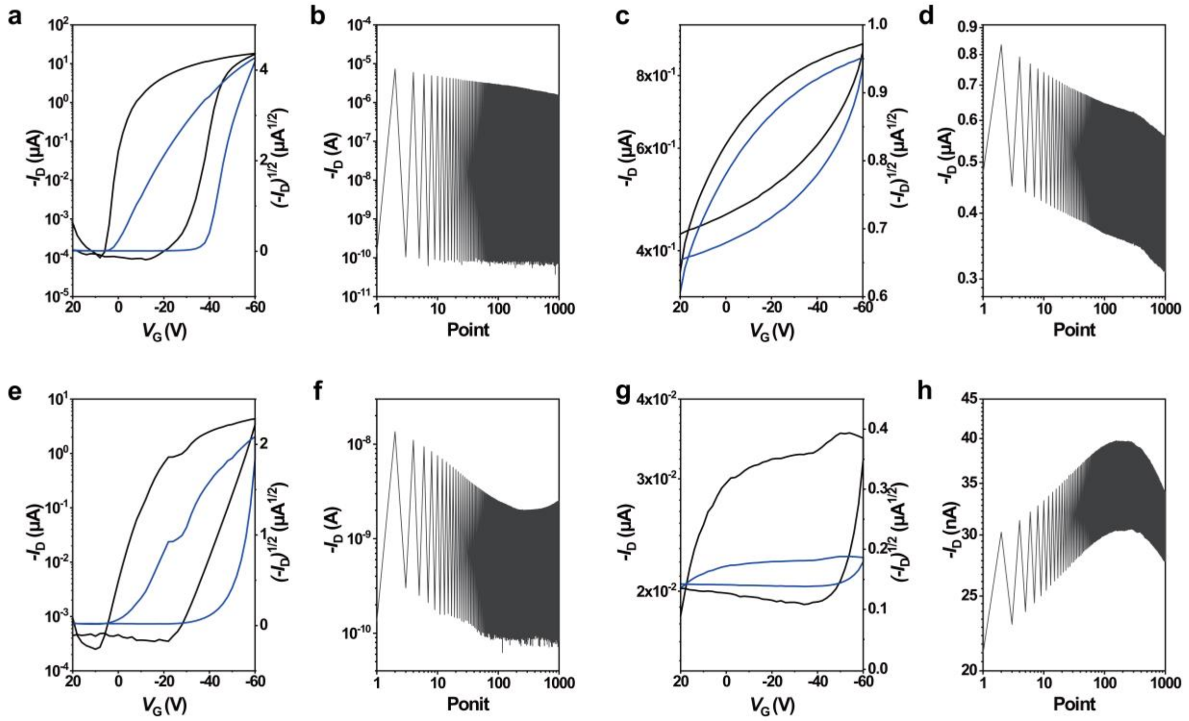

Supplementary Figure 13. The characteristics of various OFETs. Dual sweep transfer characteristic and continuous electrical test (recorded at V_D = -60 V, with V_G switching from 10 V to -60 V) for BGTC OFET based on **1-*P*** film/DPA crystal at dark state (**a, b**) and under 556 nm illumination (**c, d**), and for TGBC OFET based on **1-*P*** film/DPA film at dark state (**e, f**) and under 556 nm illumination (**g, h**).

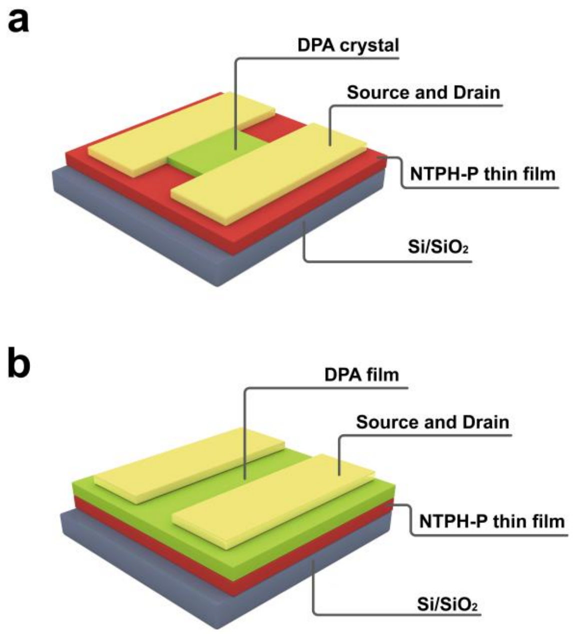

Supplementary Figure 14. Architecture for BGTC OFETs. Architecture for BGTC OFETs based on NTPH-P film and DPA crystal (**a**) and NTPH-P film and DPA film (**b**).

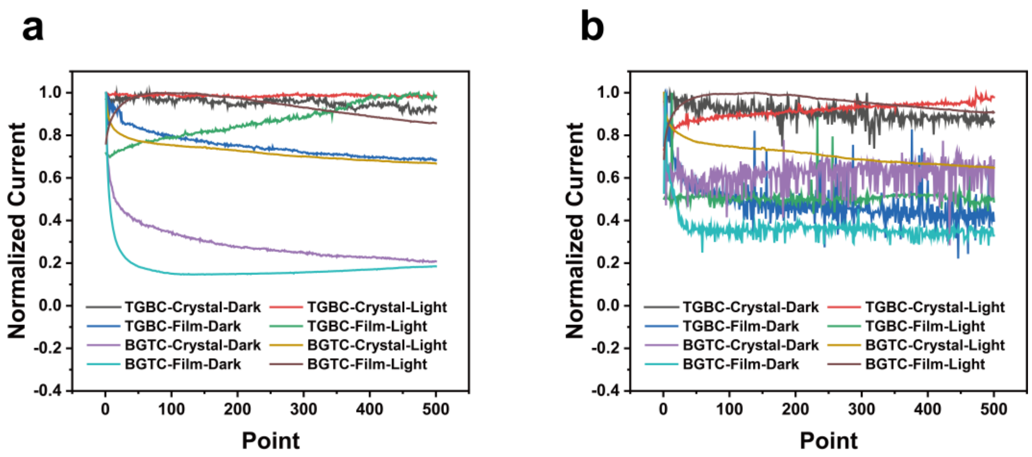

Supplementary Figure 15. Stability of various devices. The on-state (a, recorded at V_G = -60 V and V_D = -60 V) and off-state (b, recorded at V_G = 10 V and V_D = -60 V) current stability of the devices.

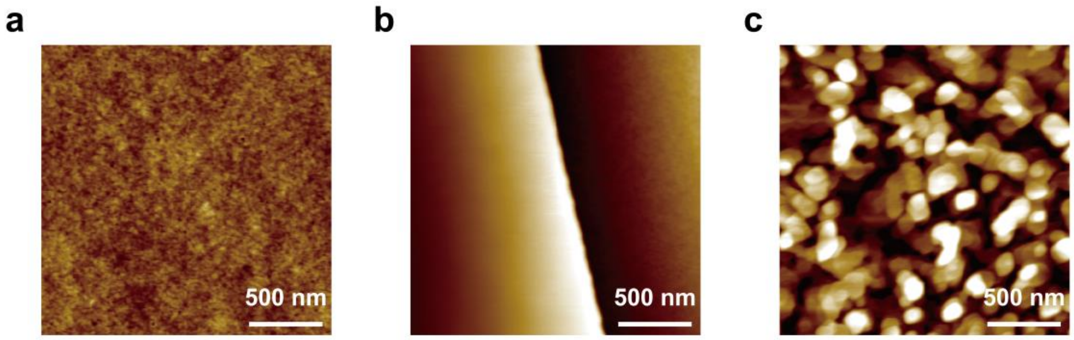

Supplementary Figure 16. AFM images of various thin films. AFM images of spin coated **1-*P*** film (a), **1-*P*** film with transferred DPA crystal (b) and **1-*P*** film with evaporated DPA thin film (c).

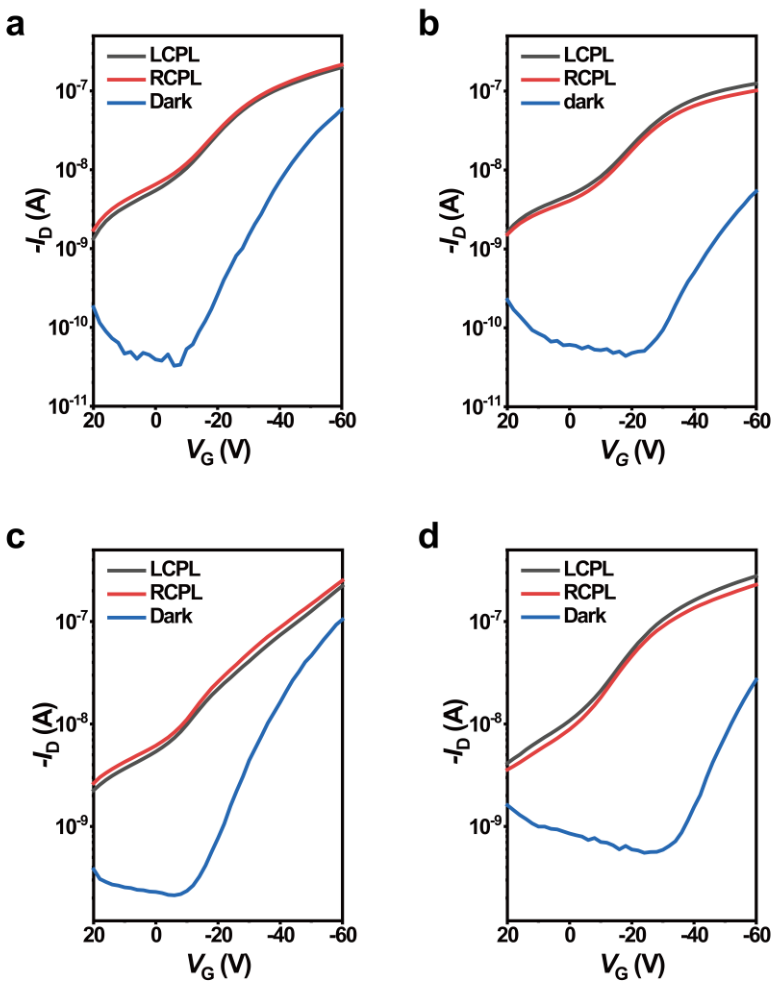

Supplementary Figure 17. The photoresponse characteristics of the photodetectors. Variation in the transfer curves of the photodetector based on **1-*P***/DPA film (**a**), **1-*M***/DPA film (**b**), **1-*P***/DPA crystal (**c**) and **1-*M***/DPA crystal (**d**) test in dark (blue line) and upon exposure to RCPL (red line) and LCPL (black line) illumination.

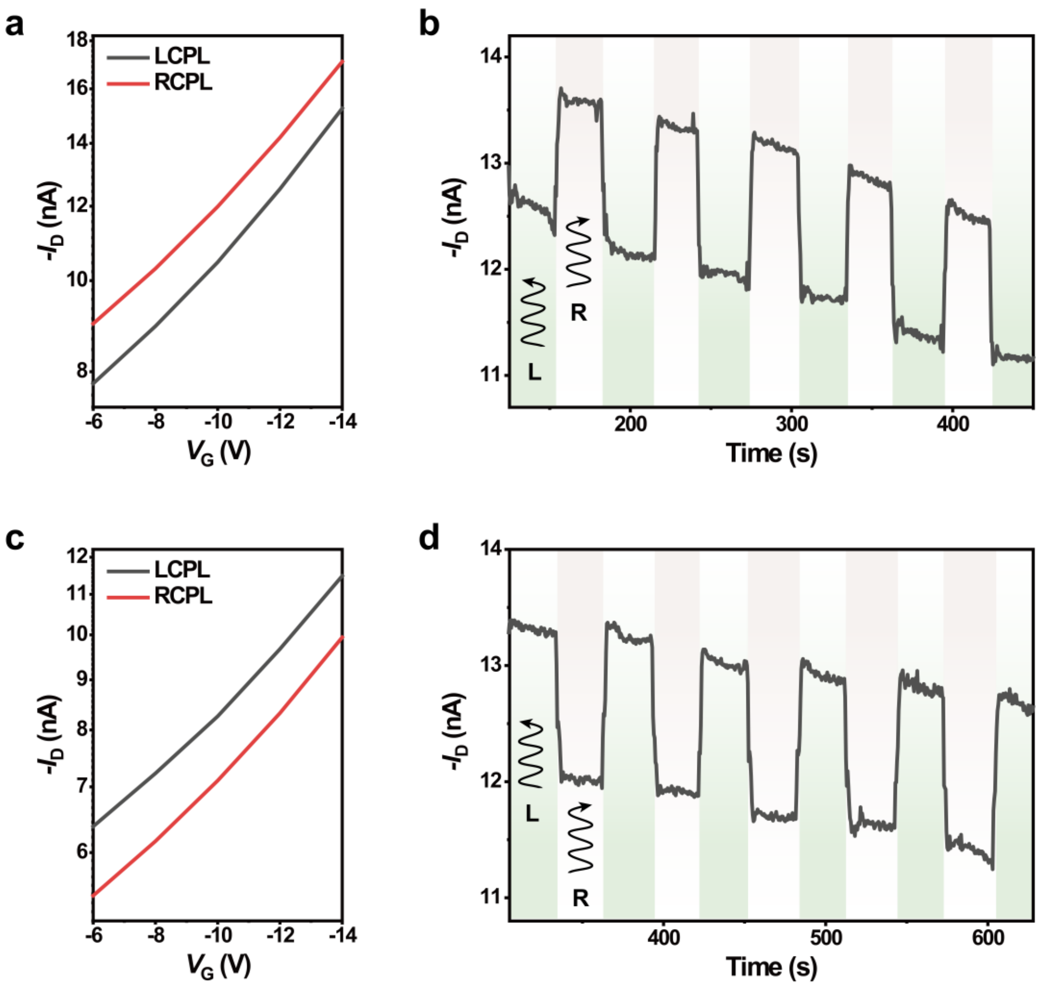

Supplementary Figure 18. The photoresponse characteristics of the photodetectors. Variation in the transfer characteristics of the photodetector based on **1-*P***/DPA film (**a**), **1-*M***/DPA film (**c**) tested upon exposure to RCPL (red line) and LCPL (black line) illumination. Dynamic I_D change of the detector in response to LCPL and RCPL illumination of the photodetector based on **1-*P*** (**b**) and **1-*M*** (**d**).

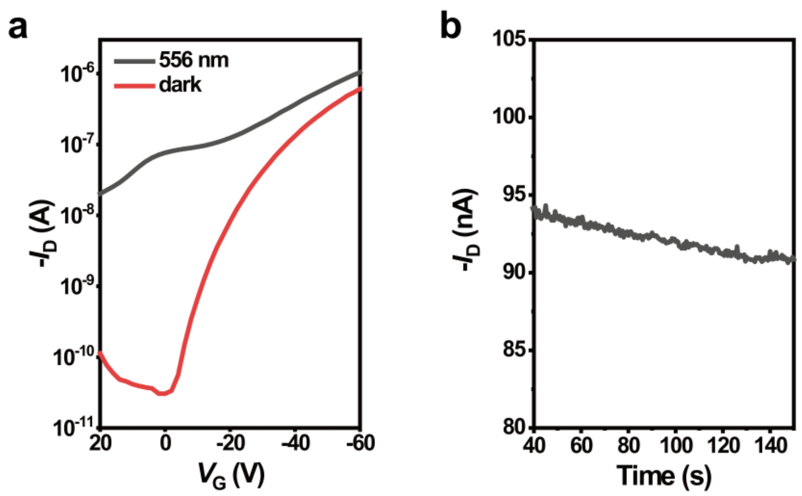

Supplementary Figure 19. The OFET characteristics of the photodetector. a, Variation in the transfer characteristics of the photodetector based on racemic NTPH-P thin film/DPA crystal. **b,** Change in drain current I_D in response to the LCPL and RCPL illumination at the wavelength of 556 nm. No obvious current change could be detected with the variation of light polarization direction.

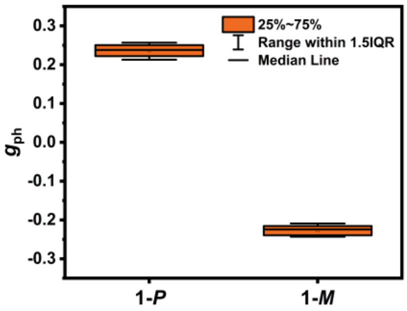

Supplementary Figure 20. Quantitative analysis result of the photodetectors. Quantitative analysis result of g_{ph} for **1-*P***/DPA crystal devices and **1-*M***/DPA crystal devices.

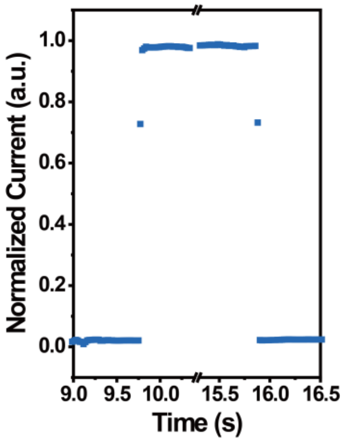

Supplementary Figure 21. Photocurrent response of the photodetector. Photocurrent response of the photodetector based on **1-*P*** film/DPA crystal.

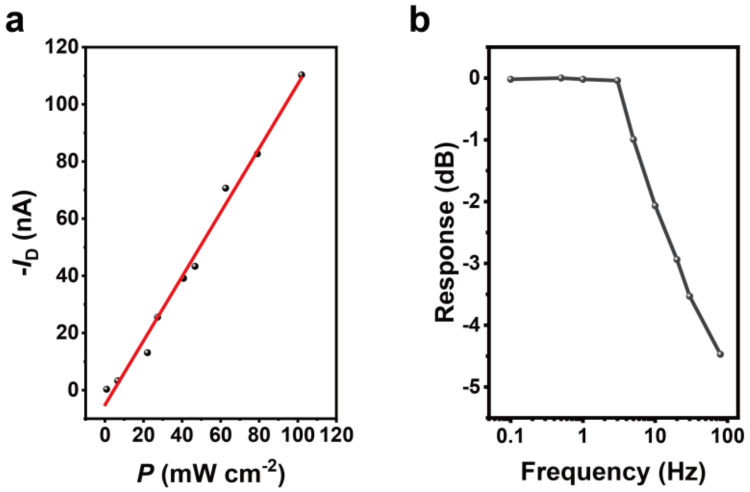

Supplementary Figure 22. The characteristics of the photodetector based on 1-*P*/DPA crystal. a, Linear dynamic range (LDR) of the of the device based on **1-*P***/DPA crystal with incident light intensity ranging from 0.8 to 101 mW cm^{-2} (V_D = -60 V, V_G = -10 V). **b,** The frequency-dependent photocurrent of the device based on **1-*P***/DPA crystal.

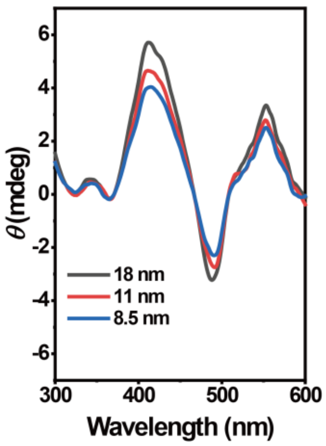

Supplementary Figure 23. Ellipticity spectrum of the chiral active layer. Ellipticity spectrum of **1-*P*** films with different thickness.

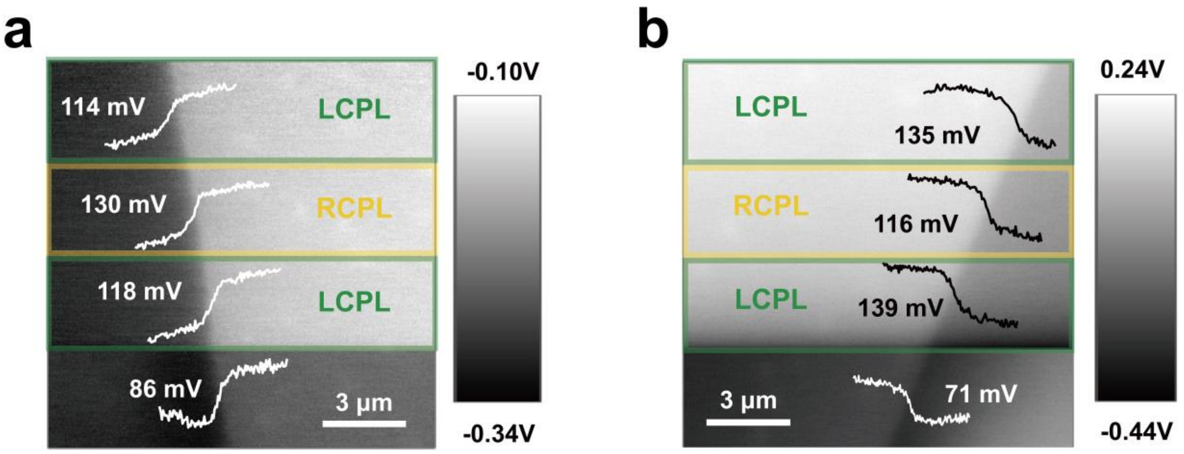

Supplementary Figure 24. SKPM captures of the heterojunctions. SKPM captures of the heterojunction interface formed by (**a**) **1-*P***/DPA crystal and (**b**) **1-*M***/DPA crystal under different CPL irradiation (from top to bottom: 556 nm RCPL, 556 nm LCPL, 556 nm RCPL, dark).

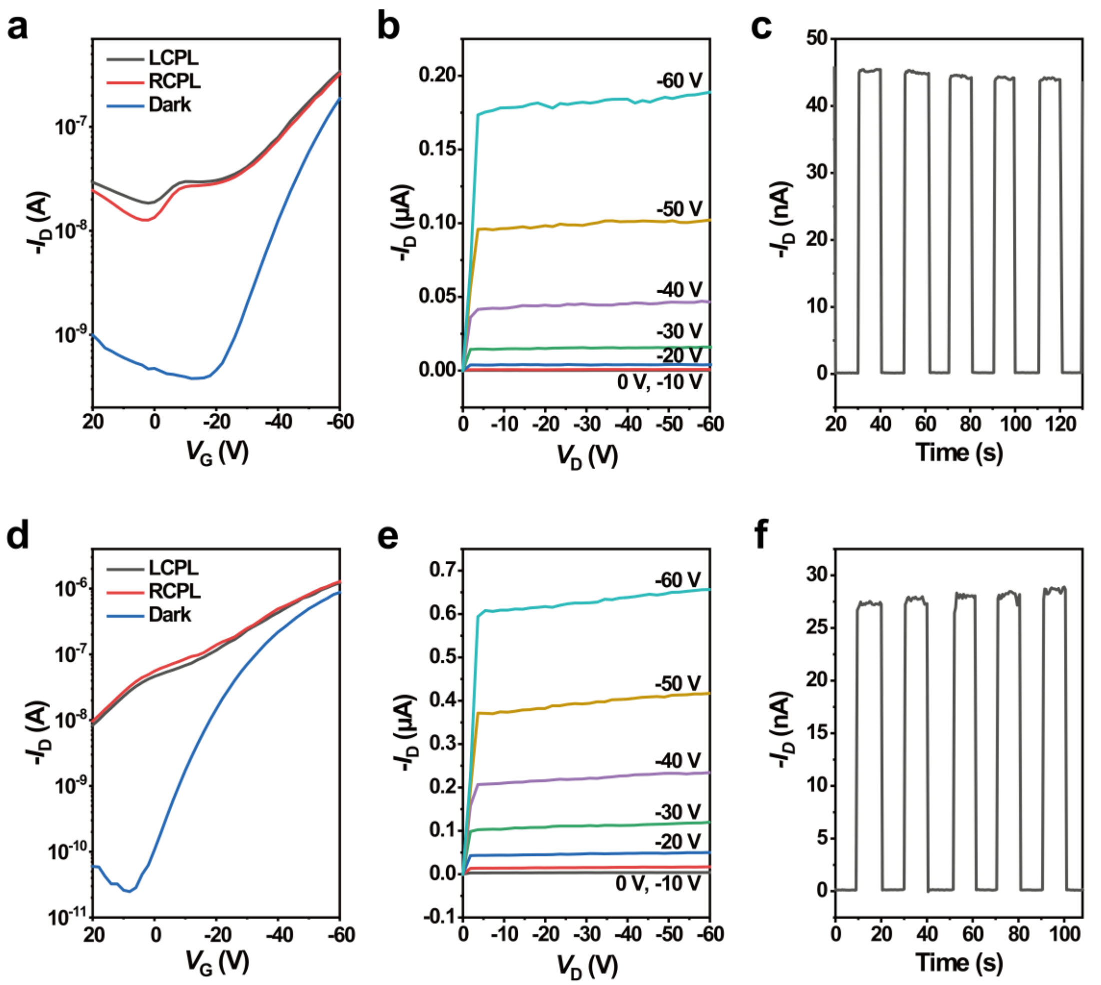

Supplementary Figure 25. The OFET characteristics of the photodetectors. Variation in the transfer characteristics of **1-*P***/DPA crystal OFET (**a**) and **1-*M***/DPA crystal OFET (**d**) test in the dark (blue line) and under RCPL (red line) and LCPL (black line) illumination at the wavelength of 488 nm. Output characteristics of the OFET based on **1-*P***/DPA crystal (**b**) and **1-*M***/DPA crystal OFET (**e**). Change in drain current I_D of the OFET based on **1-*P***/DPA crystal (**c**) and **1-*M***/DPA crystal OFET (**f**) upon repeated on–off modulation at the wavelength of 488 nm at V_G = -10 V, V_D = -60 V.

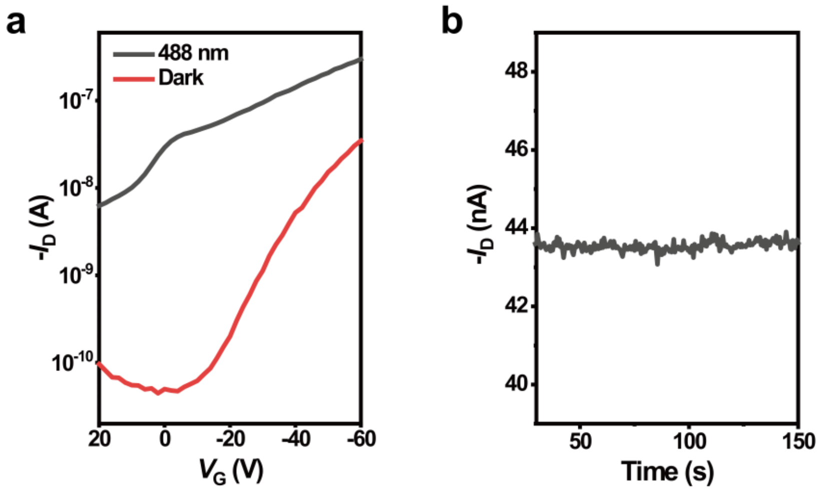

Supplementary Figure 26. The OFET characteristics of the photodetector. a, Variation in the transfer characteristics of the photodetector based on racemic NTPH-P thin film/DPA crystal. **b,** Change in I_D of the OFET in response to the LCPL and RCPL illumination at 488 nm. No obvious current change could be detected with the variation of light polarization direction.

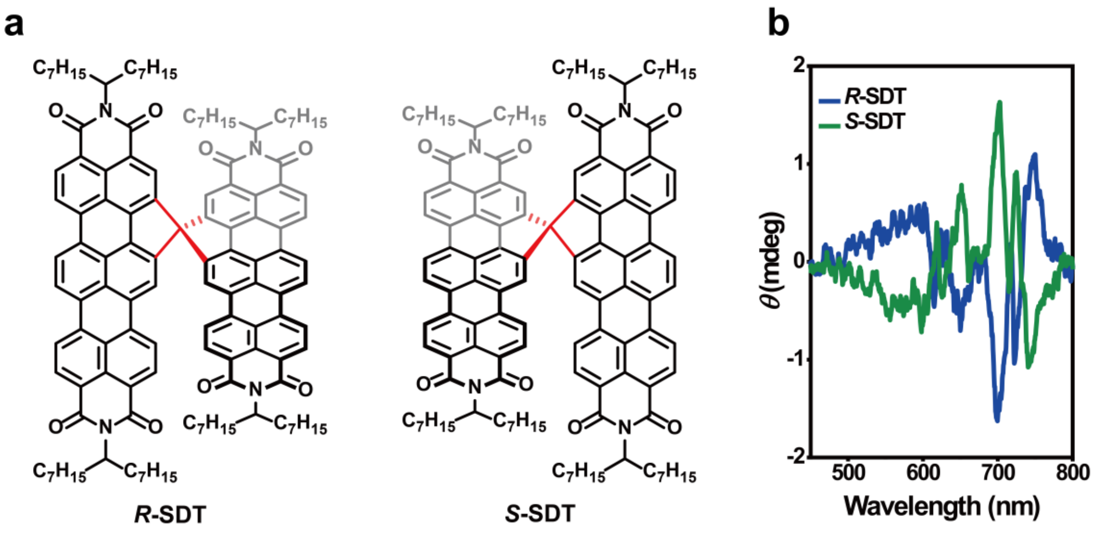

Supplementary Figure 27. Molecular structure and ellipticity spectrum of SDT. Molecular structure of the two chiral forms of the organic semiconductors SDT (**a**) and the ellipticity spectrum (**b**) of SDT thin films on quartz substrate.

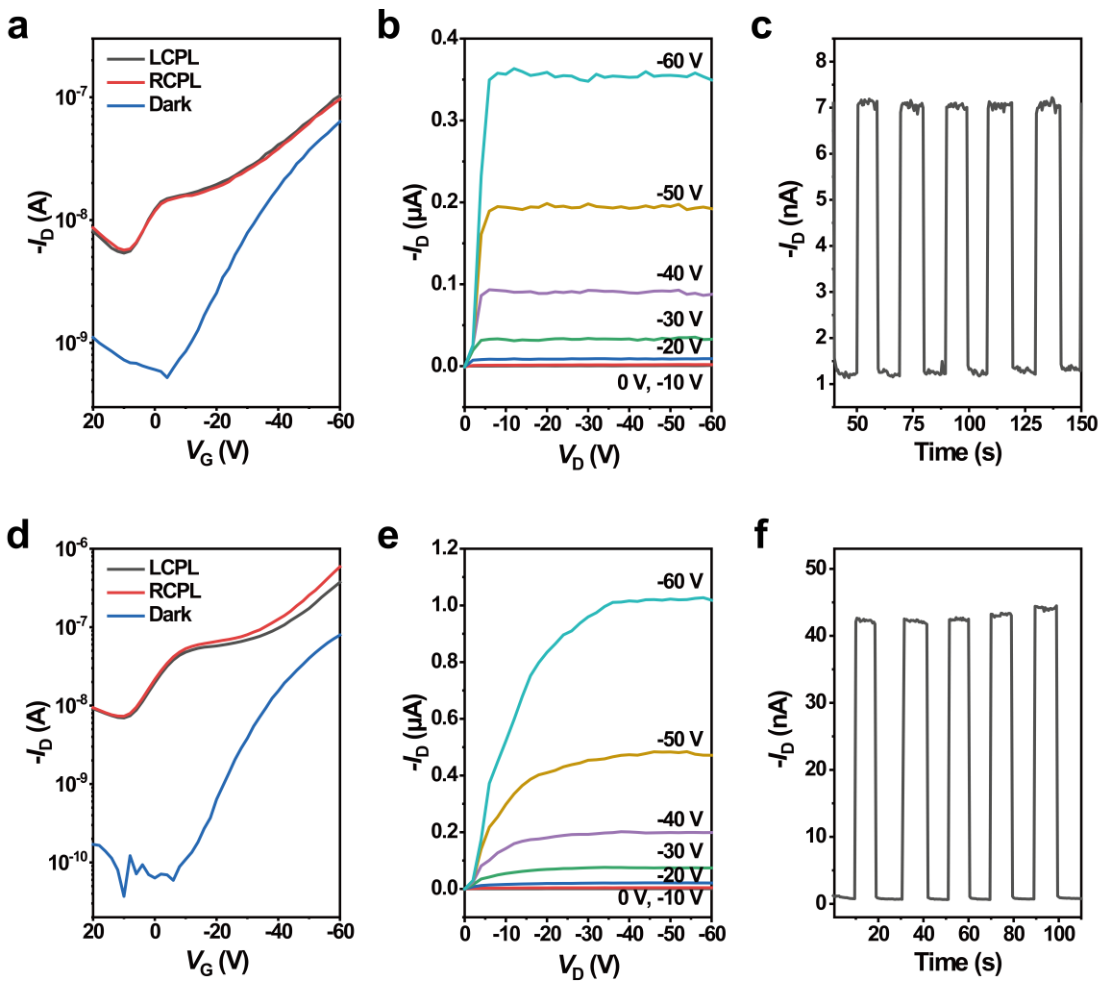

Supplementary Figure 28. The OFET characteristics of the photodetectors. Variation in the transfer characteristics of ***R*-SDT**/DPA crystal OFET (**a**) and ***S*-SDT**/DPA crystal OFET (**d**) test in the dark (blue line) and under RCPL (red line) and LCPL (black line) illumination at 700 nm. Output characteristics of the OFET based on ***R*-SDT**/DPA crystal (**b**) and ***S*-SDT** /DPA crystal OFET (**e**). Change in drain current I_D of the OFET based on ***R*-SDT**/DPA crystal (**c**) and ***S*-SDT**/DPA crystal OFET (**f**) upon repeated on–off modulation at the wavelength of 700 nm at V_G = -10 V, V_D = -60 V.

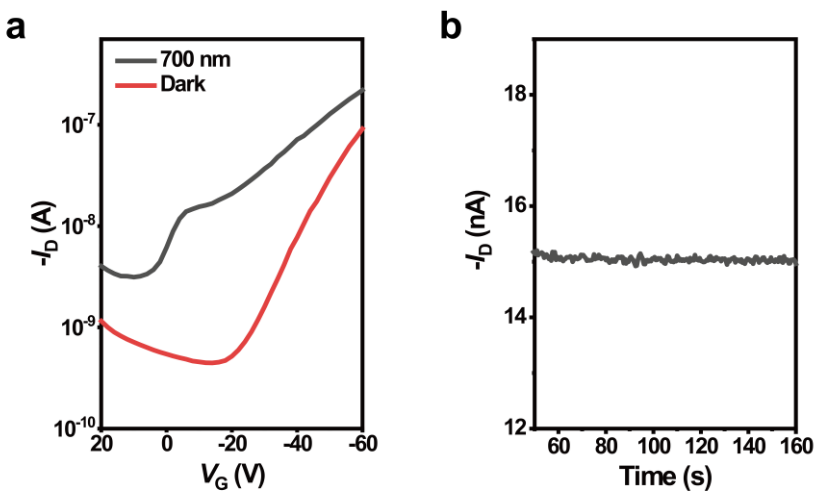

Supplementary Figure 29. The OFET characteristics of the photodetector. a, Variation in the transfer characteristics of the photodetector based on racemic SDT thin film and DPA crystal. **b,** Change in drain current I_D of the OFET in response to the LCPL and RCPL illumination at the wavelength of 700 nm. No obvious current change could be detected with the variation of light polarization direction.

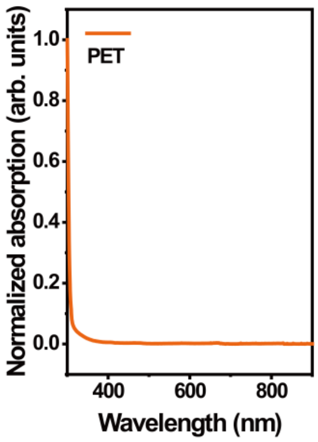

Supplementary Figure 30. Absorption spectrum of the flexible substrate. Absorption spectrum of polyethylene terephthalate (PET) substrate.

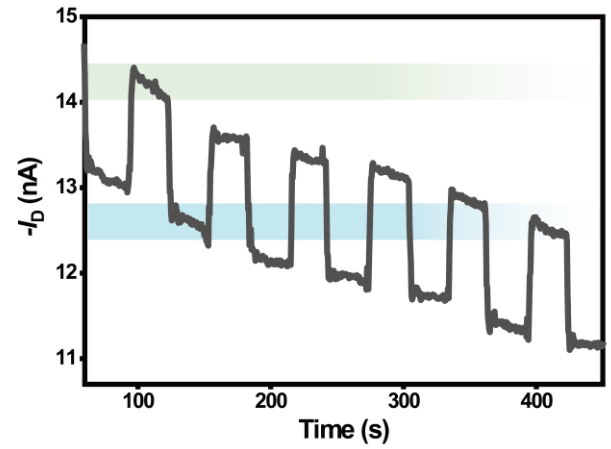

Supplementary Figure 31. Change in drain current I_D of the OFET in response to the LCPL and RCPL illumination. Dynamic I_D change of the photodetector based on **1-*P***/DPA film in response to alternating LCPL and RCPL illumination. The green shading is the range of ‘1’ and the blue shading is the definition range of ‘0’.

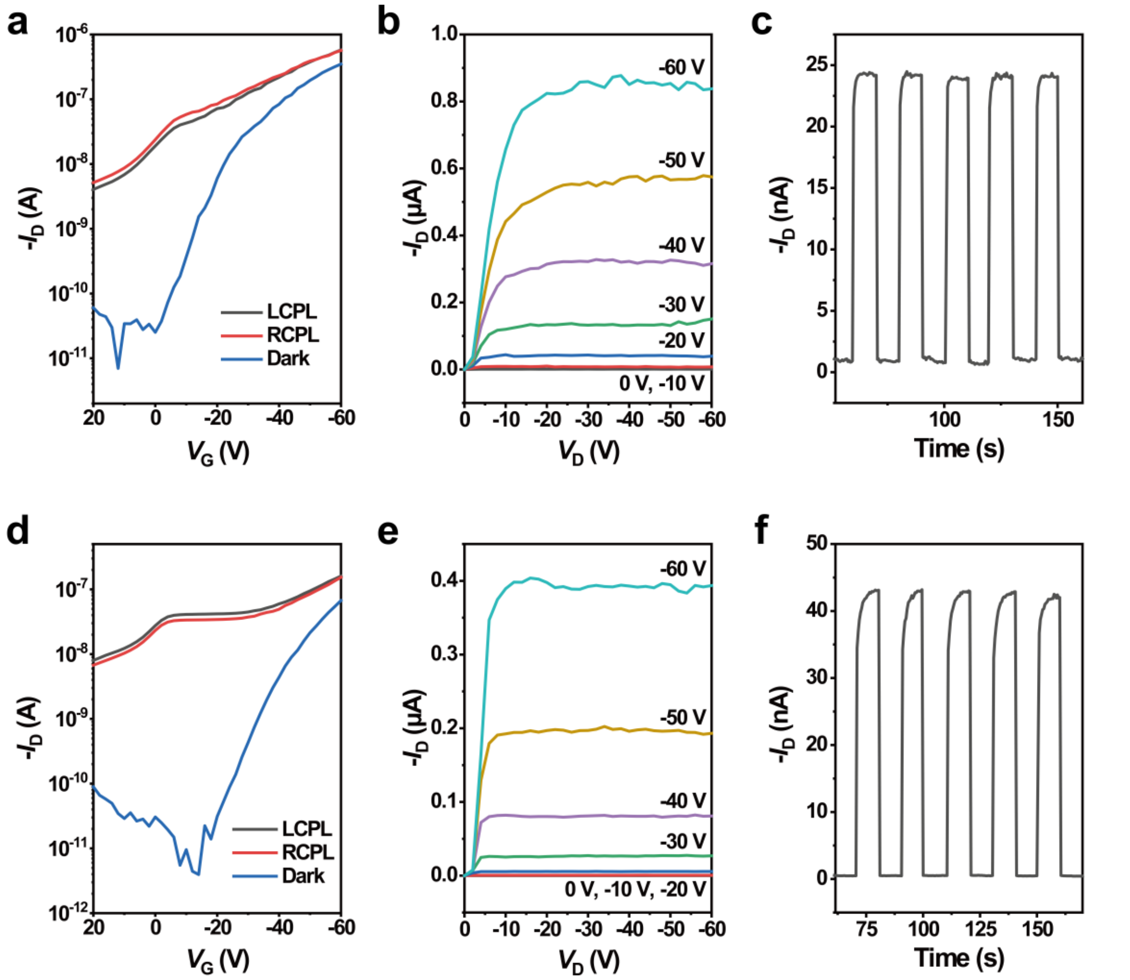

Supplementary Figure 32. The OFET characteristics of the photodetectors. Variation in the transfer characteristics of **1-*P***/DPA crystal OFET based on PET (**a**) and **1-*M***/DPA crystal OFET based on PET (**d**) test in the dark (blue line) and under RCPL (red line) and LCPL (black line) illumination at the wavelength of 556 nm. Output characteristics of **1-*P***/DPA crystal OFET based on PET (**b**) and **1-*M***/DPA crystal OFET based on PET (**e**). Change in drain current I_D of **1-*P***/DPA crystal OFET based on PET (**c**) and **1-*M***/DPA crystal OFET based on PET (**f**) upon repeated on–off modulation at the wavelength of 488 nm at V_G = -10 V, V_D = -60 V.

Supplementary Tables

Supplementary Table 1. Relevant parameters of recently published results based on chiral organic semiconductors or structure

Reference	Chiral materials	Wavelength (nm)	***P***	***R*** ($A\ W^{-1}$)	***D**** (Jones)	EQE (%)	***g***	Response speed
Nature Photonics **2013**, 7, 634-638[1]	1-Aza[6]helicene	365	-	0.01	-	-	-	2.6 ms (rise)
Adv. Mater. **2017**, 29, 1605828[2]	CPDI-Ph Nanowires	460	7.6×10^{14}	334	-	8.8×10^{4}	-	-
Adv. Funct. Mater. **2019**, 29, 1900684[3]	ProSQ-C6/PCBM	543	-	0.054	3.3×10^{14}	22	0.1	-
Adv. Funct. Mater. **2019**, 29,1808668[4]	P3CT/BN hybrid film	375	-	-	-	-	0.1	-
J. Mater. Chem. C **2020**, 8, 9271-9275[5]	P6BT (induced by CP light)	450	40.3	1.9×10^{-5}	7.4×10^{7}	1.6×10^{-3}	1.94	-
ACS Nano **2020**, 14, 14146-14156[6]	C1CPDI-Ph supramolecular single crystal	495	1122	1.7×10^{7}	2.1×10^{16}	3.0×10^{5}	0.12	~50 ms (rise&fall)
Appl. Phys. Lett. **2020**,	Chiral polythiophene	532	-	-	1.3×10^{11}	-	0.094	-

116, 053301[7]	nanowires							
Nat. Commun. **2021**, 12, 142[8]	ortho-π-Extended PDI double-[7] heterohelicence	730	2.5	0.45	2.1×10^{10}	89	0.057	-
Adv. Mater. **2021**, 33, 2004115[9]	Bis[60]phenyl-C61-butyric acid methyl ester	405	-	-	-	-	1.27	~43 ms (rise&fall)
Adv. Optical Mater. **2021**, 10, 2101044[10]	F8T2/[*M*&*P*]-aza[6]H	473	-	-	-	8.4	0.4	-
ACS Materials Lett. **2022**, 4, 2, 401-409[11]	DPPP6T/PCBM	606	-	4.4×10^{-2}	2.6×10^{10}	-	0.17	472 µs (rise) 28 µs (fall)

Supplementary Table 2. Relevant parameters of recently published results based on chiral insulating materials and chiral plasmonic metamaterials

Reference	Chiral materials	Wavelength (nm)	***P***	***R*** (A W^{-1})	***D**** (Jones)	EQE (%)	***g***	Response speed
Adv. Funct. Mater. **2019**, 29, 1805279[12]	Chiral cellulose nanocrystal (CNC) films	405	-	-	-	-	1.3	-
Adv. Funct. Mater. **2020**, 30, 2006236[13]	Cholesteric liquid crystal network (CLCN) films	830	-	300	-	≈10^4	1.9	-
		920	-	113	-			
Nat. Commun. **2015**, 6, 837[14]	Chiral plasmonic meta-molecule array	1340	-	1.5×10^{-3}	-1	-	0.72	-
Sci. Rep. **2016**, 6, 19580[15]	Metal-oxide nano helices array	635	-	-	-	-	0.16	-
Phys. Scr. **2019**, 94, 085501[16]	'Y'-shaped gold (Au) antenna	1550	-	2.7×10^{-3}	-	-	1.27	-
Nanoscale **2020**,12,5906-5913[17]	Chiral polarimeter metasurface and 2D semiconductor	790	-	2.5×10^{-3}	-	-	0.38	<100 ms (rise&fall)

Supplementary Table 3. Relevant parameters of recently published results based on chiral hybrid perovskite

Reference	Chiral materials	Wavelength (nm)	*P*	*R* (A W^{-1})	*D** (Jones)	EQE (%)	*g*	Response speed
ACS Nano **2019,** 13, 3659-3665[18]	(*R&S*-MBA)$_2$PbI$_4$ MBA=$C_6H_5C_2H_4NH_3$	518	14	0.45	2.2×10^{11}	-	-	~100 ms (rise&fall)
ACS Nano **2019**, 13, 9473-9481[19]	(*R&S*)-α-(PEA)$_2$PbI$_4$ PEA = $C_8H_9NH_3$	520	124	-	-	-	0.274	22 ms (rise) 34 ms (fall)
Nat. Commun. **2019**, 10, 1927[20]	(*R&S*-α-PEA)PbI$_3$ PEA=phenylethylamine	395	-	0.80	7.1×10^{11}	-	0.1	-
Sci. Adv. **2020**, 6, eabd3274[21]	(*R&S* -NEA)$_a$PbI$_b$ NEA=(1-naphtyl)ethylamine	395	-	2.8×10^{-4}	-	87.5	1.90	-
Angew. Chem. Int. Ed. **2020**, 59, 6442-6450[22]	[(*R&S*)-b-MPA]$_2$MAPb$_2$I$_7$ MPA=methylphenethylammonium MA=methylammonium	532	-	3.8	1.1×10^{12}	882	0.11	1.6 ms (rise) 2.1 ms (fall)
ACS Cent. Sci. **2021**,7,1261-1268[23]	[(*R&S*)-MPA]$_2$MAPb$_2$I$_7$/MAPbI$_3$ MPA=methylphenethylamine,	520	10^5	1.2×10^{-3}	1.1×10^{12}	2.8	0.67	2 ms (rise) 2.4 ms (fall)

	MA=methylammonium							
J. Am. Chem. Soc. **2021**, 143, 8437-8445[24]	(*R&S*-α-PEA)$_2$PbI$_4$ PEA=phenylethylamine	505	-	47.1	1.2×10^{13}	-	0.15	-
ACS Nano **2021**, 15, 7608-7617[25]	(*R&S*-MBA)$_2$CuCl$_4$/SWCNT MBA=methylbenzylammonium	405	-	452	-	-	0.25	-
Angew. Chem. Int. Ed. **2021**, 60, 8415-8418[26]	[(*R&S*)-β-MPA], MPA=methylphenethylammonium	520	-	1.2×10^{-6}	1.2×10^{7}	-	0.23	-
ACS Nano **2021**, 15, 588-595[27]	(*R&S*-MBA)$_2$PbI$_4$ MBA=$C_6H_5C_2H_4NH_3$	486	-	-	-	-	0.1	-
J. Am. Chem. Soc. **2021**, 143, 14077-14082[28]	(*R&S*-BPEA)$_2$PbI$_4$ BPEA=1-(4-bromophenyl)ethylammonium	520	2.0×10^{4}	2.1×10^{-3}	3.0×10^{11}	-	0.13	-

209

Supplementary Table 4. Architecture, mobility and test condition of recently published results based on chiral organic semiconductors or structure

Reference	Chiral materials	Architecture	Charge transport property	Device performance	Test condition
Nature Photonics **2013**, 7, 634-638[1]	1-Aza[6]helicene	Thin film FET	10^{-4} $cm^2V^{-1}s^{-1}$ (mobility)	Transfer curve	Nitrogen condition
Adv. Mater. **2017**, 29, 1605828[2]	CPDI-Ph Nanowires	NWs FET	6.2×10^{-2} $cm^2V^{-1}s^{-1}$ (mobility)	Transfer curve	Vacuum chamber
Adv. Funct. Mater. **2019**, 29, 1900684[3]	ProSQ-C6/PCBM	OPV	-	J_{sc}	Nitrogen condition
Adv. Funct. Mater. **2019**, 29,1808668[4]	P3CT/BN hybrid film	Photodiode	-	Real-time current	Ambient condition
J. Mater. Chem. C **2020**, 8, 9271-9275[5]	P6BT (induced by CPL)	Thin film FET	3.5×10^{-3} $cm^2V^{-1}s^{-1}$ (mobility)	Transfer curve	Ambient condition
ACS Nano **2020**, 14, 14146-14156[6]	C1CPDI-Ph supramolecular	SC FET	1.0 $cm^2V^{-1}s^{-1}$ (n-doping mobility) 4.9×10^{-3} $cm^2V^{-1}s^{-1}$ (intrinsic mobility)	Real-time current	Nitrogen condition
Appl. Phys. Lett. **2020**, 116, 053301[7]	Chiral polythiophene nanowires	OPV	-	Real-time current	-

Nat. Commun. **2021**, 12, 142[8]	ortho-π-Extended PDI double-[7] heterohelicence	Thin film FET	1.7×10^{-3} cm^{2}V^{-1}s^{-1} (mobility)	Real-time current	Vacuum chamber
Adv. Mater. **2021**, 33, 2004115[9]	Bis[60]phenyl-C61-butyric acid methyl ester	Thin film FET	1.4×10^{-5} cm^{2}V^{-1}s^{-1} (mobility)	Real-time current	-
Adv. Optical Mater. **2021**, 10, 2101044[10]	F8T2/[*M*&*P*]-aza[6]H	OPV	-	Real-time current	-
ACS Materials Lett. **2022**, 4, 2, 401-409[11]	DPPP6T/PCBM	OPV	-	J_{sc}	-
Our work	NTPH-P/DPA	Heterojunction FET	1.1 cm^{2}V^{-1}s^{-1} (mobility)	Real-time current	Ambient condition

Supplementary Table 5. The contact resistance of different devices by YFM

Device	R_C (MΩ) at V_G = -56 V
DPA crystal TGBC OFET	0.713
**1-*P***/DPA crystal-based TGBC OFET	44.267

Supplementary Table 6. The decay of the on-state current of the four kinds of devices

Device	Dark	Under illumination (556 nm)
**1-*P***/DPA crystal-based TGBC OFET	4%	3%
**1-*P***/DPA film-based TGBC OFET	30%	31%
**1-*P***/DPA crystal-based BGTC OFET	130%	39%
**1-*P***/DPA film-based BGTC OFET	137%	33%

Supplementary Table 7. Related parameters (*g*, *R*, EQE, *D) of different devices**

Device	g	R (A W^{-1})	EQE (%)	D^* (Jones)
**1-*P***/DPA crystal 556 nm	+0.247	0.233	51.9	1.33×10^{9}
**1-*M***/DPA crystal 556 nm	-0.238	0.279	59.5	1.23×10^{9}
**1-*P***/DPA film 556 nm	+0.119	6.97×10^{-3}	15.6	3.27×10^{8}

**1-*M***/DPA film<br>556 nm	-0.115	6.05×10^{-3}	13.5	2.32×10^{8}
**1-*P***/DPA crystal<br>488 nm	+0.151	0.125	31.9	5.13×10^{8}
**1-*M***/DPA crystal<br>488 nm	-0.150	0.121	30.6	1.01×10^{9}
***R*-SDT**/DPA crystal<br>700 nm	+0.117	0.220	39.1	4.82×10^{9}
***S*-SDT**/DPA crystal<br>700 nm	-0.116	0.206	36.5	3.98×10^{9}
**1-*P***/DPA crystal<br>flexible-556 nm	+0.199	0.123	27.5	3.56×10^{8}
**1-*M***/DPA crystal<br>flexible-556 nm	-0.209	0.114	25.3	6.94×10^{8}

Supplementary Table 8. Fluorescence lifetime of DPA on different substrates

Substrate	Lifetime (ns)
Silica	1.590
CYTOP	2.178
**1-*P*** film	1.062

Supplementary Notes

Supplementary Note 1: OFET fabrication.

Chiral molecules NTPH-P and SDT were prepared as previously reported, and separated using preparative chiral high-performance liquid chromatography (HPLC). Bilayer donor-acceptor junction OFETs were fabricated on quartz substrate. The substrates were cleaned with pure water, then piranha solution (H_2SO_4/H_2O_2 = 7:3), pure water and finally with pure isopropyl alcohol, and then blow-dried with high-purity nitrogen gas, and then 2 nm chromium (Cr) and 20 nm gold (Au) were evaporated on the substrate through a shadow mask as source and drain electrodes. The chiral active layers were prepared by spin-coating from a 4 $mgmL^{-1}$ solution in chloroform-n-hexane. The crystal-based devices were fabricated by using cantilever probes to mechanically transfer DPA crystals onto the chiral active layer directly above the channel. DPA crystals were achieved by means of physical vapor transport (PVT) (175°C, 2 h, 30 Pa, under argon atmosphere). DPA thin films were fabricated by evaporation in high vacuum chamber under a pressure of 6×10^{-4} Pa, and the deposition rates were monitored using a quartz oscillating crystal and controlled at 0.1-0.2 Å s^{-1}. CYTOP (3:1) solution was spin-coated at 2000 rpm for 30 s, and annealed at 90°C for 20 min to fabricate dielectric layer. And then 40 nm aluminum (Al) was evaporated as the gate electrode.

The fabrication of the flexible device based on PET substrate is similar to that of the rigid device. The only difference is that during the preparation of the dielectric layer, the annealing condition is 75°C for 30 min to reduce the deformation of flexible substrates. Other procedures of device fabrication are the same as those on quartz substrates.

The process of device construction has been shown in Supplementary Figure 6 and 7.

Supplementary Note 2: Optical properties of the CPL detector.

The ellipticity θ of materials could be calculated according to:

$$\theta = \arctan\left(\frac{e^{CD} - 1}{e^{CD} + 1}\right) \quad (1)$$

For CD values<0.5 rad the usual approximation for ellipticity:$\theta = \frac{CD}{2}$[29].

To distinguish the dissymmetry factor of absorption and photocurrent, we introduce the $\boldsymbol{g}_{\mathbf{abs}}$ and $\boldsymbol{g}_{\mathbf{ph}}$ respectively. The absorption dissymmetry factor $\boldsymbol{g}_{\mathbf{abs}}$ can be obtained according to the equation:

$$g_{abs} = \frac{\Delta A}{A} \quad (2)$$

where ΔA is the differential absorption of LCPL and RCPL, A is the average absorption of LCPL and RCPL.

In order to ensure the stability of the chiroptical properties of the pair of enantiomers, ellipticity spectra are demonstrated under various conditions. Compared to the signal in solution, a slight redshift in the thin film is observed, which has little effect on the choice of the detection wavelength (Supplementary Figure 1). Owing to the various treatments during the device fabrication process, such as vacuum evaporation and annealing, the vacuum stability and thermal stability also need to be demonstrated. It can be concluded from Supplementary Figure 2 that the ellipticity and $\boldsymbol{g}_{\mathbf{abs}}$ are basically unaffected (Supplementary Figure 2). Especially, to prove the uniformity of the thin film, the ellipticity and $\boldsymbol{g}_{\mathbf{abs}}$ spectra of the chiral film with different azimuthal rotations are also demonstrated. At 488 nm and 556 nm, the fluctuation of $\boldsymbol{g}_{\mathbf{abs}}$ is around 3-5%, which may mainly originate from the measurement (Supplementary Figure 3).

Supplementary Note 3: Optoelectronic properties.

To prove the unique merits of the detector, NTPH-P film based OFETs and DPA crystal based OFETs were tested under light illumination at the wavelength of 556 nm, and the current barely change under different light conditions (Supplementary Figure 4). These results indicate that, the photoresponse originated from the donor-acceptor junction based on chiral molecule and semiconductor.

As for the CPL detector, DPA in thin film and single crystal morphologies are both employed as functional layers in CPL detectors. In order to compare the stability of the detectors in air, four kinds of devices are fabricated for comparison: **1-*P***/DPA crystal-based TGBC OFET, **1-*P***/DPA film-based TGBC OFET (Supplementary Figure 9), **1-*P***/DPA crystal-based bottom-gate top-contact (BGTC) OFET (Supplementary Figure 14a), **1-*P***/DPA film-based BGTC OFET (Supplementary Figure 14b). The decay of the on-state current (V_G=-60V) of the detector in the dark state are 4%, 30%, 130% and 137%, respectively. While the values are 3%, 31%, 39%, 33% under the illumination of 556 nm (84.88 mW cm^{-2}) (Supplementary Figure 12-13). The long-term current stability of the novel detector structure might mainly benefit from the low-trap density at the CYTOP/DPA interface.

Supplementary Note 4: Y-function method for contact resistance.

The contact resistance of the device was calculated according to the method of Y-function method (YFM) which based on dedicated expression of transfer characteristics $I_D(V_G)$.[30,31] Herein, the devices used for comparison are DPA crystal based TCBG FET and **1-*P***/DPA crystal based TCBG FET. The contact resistance of the devices is extracted from transfer characteristics in a linear regime ($V_G - V_T \gg V_D$, where V_G, V_T and V_D are gate voltage, threshold voltage and drain voltage), with the equation of I_D as follows:

$$I_D = (W/L)\mu C_i(V_G - V_T)V_D = [(W/L)\mu_0 C_i(V_G - V_T)V_D]/[1 + \alpha(V_G - V_T)] \quad (3)$$

where W and L are channel width and length, μ and μ_0 are effective mobility and intrinsic mobility, C_i is the unit area capacitance of the dielectric, α is the mobility attenuation factor, respectively. The mobility attenuation factor is defined as follows:

$$\alpha = \alpha_{ch} + \mu_0 C_i R_c(W/L) \approx \mu_0 C_i R_c(W/L) \quad (4)$$

where α_{ch} and R_c are channel contribution on attenuation factor and the contact resistance.

Y-function is founded by I_D and g_m (transconductance) as follows:

$$Y = \frac{I_D}{\sqrt{g_m}} = \frac{I_D}{\sqrt{\left(\frac{\partial I_D}{\partial V_G}\right)}} \approx \sqrt{(W/L)\mu_0 C_i V_D}(V_G - V_T) \quad (5)$$

And the contact resistance can be calculated as:

$$R_C \approx V_D \times \left(\frac{\partial Y}{\partial V_G}\right)^{-1} \times \frac{\partial\left(1/\sqrt{g_m}\right)}{\partial V_G} \quad (6)$$

Supplementary Note 5: Mechanism of the photoresponse

The photocurrent dissymmetry factor $\boldsymbol{g}_{\mathbf{ph}}$ of the CPL photo FET is two orders of magnitude larger than $\boldsymbol{g}_{\mathbf{abs}}$. The mainly amplification effect may originate from the variation of the photoinduction electric field of the device under the illumination of LCPL and RCPL. The main influencing factor of the dissymmetry is the photoinduction electric field which originated from the electrons trapped and accumulated in the chiral active layer. From the SKPM in Figure 3f, the potential drop at the donor-acceptor junction of **1-*P*** film/DPA crystal at dark state, LCPL irradiation and RCPL irradiation are 54.5 mV, 76.1 mV and 89.4 mV, respectively. Compared to the dark state, the increase of the potential drop under the illumination could reflect the photoinduction electric field at the donor-acceptor junction. Hence, the total electric field of the device under the illumination is the sum of the gate electric field (E_G) and photoinduction electric field, which will induce the current of the device.

According to the equation of the electric field (E, $E=U/d$), during the measurement of the photocurrent dissymmetry, V_G is fixed to -10 V and the thickness of the dielectric layer is around 500 nm, so E_G is calculated around 2.0×10^{7} V m^{-1}. In our devices, the electrons mainly accumulate in the chiral active layer with a few molecular thicknesses owing to the poor charge transport property of the chiral active layer. Here, the single crystal structure is unknown owing to the poor crystallinity of the chiral molecule, which leads to the loss of the tilted angel information of the chiral molecule on the substrate. In order to qualitatively analyze the effect of built-in potential to photocurrent dissymmetry, we assume the thickness of the accumulation layer is 3 nm. Then, the photoinduction electric field of the device under LCPL and RCPL could be calculated around 7.2×10^{6} V m^{-1} and 1.2×10^{7} V m^{-1}, respectively. Hence, the total electric field of the device under LCPL and RCPL are around 2.7×10^{7} V m^{-1} and 3.2×10^{7} V m^{-1}, which could be converted to a gate voltage of -13.5 V and -16.0 V under the dark state. The I_D at V_G = -13.5 V and V_G = -16 V are 3.16×10^{-10} A and 4.17×10^{-10} A of the **1-*P***/DPA crystal based TGBC photo FET in Figure 1c, respectively. The dissymmetry factor for the approximate current is 0.28, which is the same order of magnitude as the photocurrent dissymmetry.

The photoinduction electric field also influence the relationship between the light intensity

or the thickness of the film and the photocurrent. Considering the electric field directly affected the photocurrent dissymmetry, we evaluated the dissymmetry factor of E:

$$g_{\mathrm{E}} = \frac{(E_{\mathrm{G}} + E_{\mathrm{LCPL}}) - (E_{\mathrm{G}} + E_{\mathrm{RCPL}})}{1/2\,[(E_{\mathrm{G}} + E_{\mathrm{LCPL}}) + (E_{\mathrm{G}} + E_{\mathrm{RCPL}})]} = \frac{(E_{\mathrm{LCPL}} - E_{\mathrm{RCPL}})}{1/2\,(2E_{\mathrm{G}} + E_{\mathrm{LCPL}} + E_{\mathrm{RCPL}})} \quad (7)$$

where E_{LCPL} and E_{RCPL} are the photoinduction electric field originated from LCPL illumination and RCPL illumination. During the measurement of the photocurrent dissymmetry, V_{G} is fixed at -10 V, so E_{G} is a constant. With the increase of the thickness or the light intensity, even if the E_{LCPL} and E_{RCPL} increase proportionally, the electric field dissymmetry $\boldsymbol{g}_{\mathbf{E}}$ still enhances according to Eq. (7). The increase of the electric field results in the increase of the photocurrent, which leads to the increase of the dissymmetry factor with the enhancement of the light intensity or the thickness of the film.

Supplementary Note 6: Information transmission.

The stability of the photocurrent is the mean factor of the further application, such as information transmission. Taking **1-*P***/DPA film based TGBC OFET as an example (Supplementary Figure 31), signals can be defined in terms of the initial photocurrent, while for LCPL is '0'and RCPL is '1'. Herein, considering the fluctuation of the current, according to the variance, the photocurrent of '1' is defined in the range of -14.21±0.39 nA, while the '0' is -12.62±0.35 nA. After 50 s, the average photocurrent for RCPL is around -13.59 nA, which is out of the definition range. What is more exaggerated, after 250 s, the average photocurrent for RCPL, which is ideally defined as '1', is -12.58 nA, which exist within the definition range of '0'. The attenuation of the absolute value of the photocurrent during the test process seriously affects the signal transmission, resulting in the invalid detection.

We use Python to process Morse cipher-based data encryption, the main process can be divided into two stages: predefinition and decoding. Due to the individual differences of devices, we need to accurately define 'dot' and 'dash' (0 and 1 in the code) in the initial phase of device testing. The interval between the signals depends on the off state, which can be divided into signal interval, character interval and sentence interval according to the length of the off-state time. In this example, the predefined time is 75 s, the interval time between signals is 0-10 s, and the interval time between characters is 10-20 s.

Supplementary References

1 Yang, Y., da Costa, R. C., Fuchter, M. J. & Campbell, A. J. Circularly Polarized Light Detection by a Chiral Organic Semiconductor Transistor. *Nat. Photonics* **7**, 634-638 (2013).

2 Shang, X. *et al.* Supramolecular nanostructures of chiral perylene diimides with amplified chirality for high-performance chiroptical sensing. *Adv. Mater.* **29** (2017).

3 Schulz, M. *et al.* Chiral Excitonic Organic Photodiodes for Direct Detection of Circular Polarized Light. *Adv. Funct. Mater.* **29**, 1900684 (2019).

4 Kim, N. Y. *et al.* Chiroptical-Conjugated Polymer/Chiral Small Molecule Hybrid Thin Films for Circularly Polarized Light-Detecting Heterojunction Devices. *Adv. Funct. Mater.* **29**, 1808668 (2019).

5 Cheng, J. *et al.* Enabling discrimination capability in an achiral F6BT-based organic semiconductor transistorviacircularly polarized light induction. *J. Mater. Chem. C* **8**, 9271-9275 (2020).

6 Shang, X. *et al.* Surface-doped quasi-2D chiral organic single crystals for chiroptical sensing. *ACS Nano* **14**, 14146-14156 (2020).

7 Wang, Z., Gao, M., Hao, X. & Qin, W. Helical-chiroptical nanowires generated orbital angular momentum for the detection of circularly polarized light. *Appl. Phys. Lett.* **116**, 053301 (2020).

8 Zhang, L. *et al.* π-Extended perylene diimide double-heterohelicenes as ambipolar organic semiconductors for broadband circularly polarized light detection. *Nat. Commun.* **12**, 142 (2021).

9 Shi, W. *et al.* Fullerene Desymmetrization as a Means to Achieve Single-Enantiomer Electron Acceptors with Maximized Chiroptical Responsiveness. *Adv. Mater.* **33**, 2004115 (2021).

10 Ward, M. D. *et al.* Highly Selective High-Speed Circularly Polarized Photodiodes Based on π-Conjugated Polymers. *Adv. Opt. Mater.* **10**, 2101044 (2022).

11 Liu, L. *et al.* Building supramolecular chirality in bulk heterojunctions enables amplified dissymmetry current for high-performing circularly polarized light detection. *ACS Materials Letters* **4**, 401-409 (2022).

12 Grey, P. *et al.* Field-Effect Transistors on Photonic Cellulose Nanocrystal Solid Electrolyte for Circular Polarized Light Sensing. *Adv. Funct. Mater.* **29**, 1805279 (2019).

13 Han, H. *et al.* High-performance circularly polarized light-sensing near-infrared organic phototransistors for optoelectronic cryptographic primitives. *Adv. Funct. Mater.* **30**, 2006236 (2020).

14 Li, W. *et al.* Circularly polarized light detection with hot electrons in chiral plasmonic

metamaterials. *Nat. Commun.* **6**, 8379 (2015).

15 Lee, S. H. *et al.* Highly photoresponsive and wavelength-selective circularly-polarized-light detector based on metal-oxides hetero-chiral thin film. *Sci. Rep.* **6**, 19580 (2016).

16 Xiao, W., Shi, X., Zhang, Y., Peng, W. & Zeng, Y. Circularly polarized light detector based on 2D embedded chiral nanostructures. *Phys. Scr.* **94**, 085501 (2019).

17 Jiang, Q. *et al.* Ultrathin circular polarimeter based on chiral plasmonic metasurface and monolayer MoSe2. *Nanoscale* **12**, 5906-5913 (2020).

18 Ma, J. *et al.* Chiral 2D Perovskites with a High Degree of Circularly Polarized Photoluminescence. *ACS Nano* **13**, 3659-3665 (2019).

19 Wang, J. *et al.* Aqueous synthesis of low-dimensional lead halide perovskites for room-temperature circularly polarized light emission and detection. *ACS Nano* **13**, 9473-9481 (2019).

20 Chen, C. *et al.* Circularly polarized light detection using chiral hybrid perovskite. *Nat. Commun.* **10**, 1927 (2019).

21 Ishii, A. & Miyasaka, T. Direct detection of circular polarized light in helical 1D perovskite-based photodiode. *Sci. Adv.* **6**, eabd3274 (2020).

22 Wang, L. *et al.* A Chiral Reduced-Dimension Perovskite for an Efficient Flexible Circularly Polarized Light Photodetector. *Angew. Chem. Int. Ed.* **59**, 6442-6450 (2020).

23 Zhang, X. *et al.* Great amplification of circular polarization sensitivity via heterostructure engineering of a chiral two-dimensional hybrid perovskite crystal with a three-dimensional MAPbI3 crystal. *ACS Central Science* **7**, 1261-1268 (2021).

24 Zhao, Y. *et al.* Chiral 2D-Perovskite Nanowires for Stokes Photodetectors. *J. Am. Chem. Soc.* **143**, 8437-8445 (2021).

25 Hao, J. *et al.* Direct Detection of Circularly Polarized Light Using Chiral Copper Chloride–Carbon Nanotube Heterostructures. *ACS Nano* **15**, 7608-7617 (2021).

26 Li, D. *et al.* Chiral lead-free hybrid perovskites for self-powered circularly polarized light detection. *Angew. Chem. Int. Ed.* **60**, 8415-8418 (2021).

27 Wang, J. *et al.* Spin-Dependent Photovoltaic and Photogalvanic Responses of Optoelectronic Devices Based on Chiral Two-Dimensional Hybrid Organic–Inorganic Perovskites. *ACS Nano* **15**, 588-595 (2021).

28 Peng, Y. *et al.* Realization of vis–NIR Dual-Modal Circularly Polarized Light Detection in Chiral

Perovskite Bulk Crystals. *J. Am. Chem. Soc.* **143**, 14077-14082 (2021).

29 Schulz, M. *et al.* Giant intrinsic circular dichroism of prolinol-derived squaraine thin films. *Nat. Commun.* **9**, 2413 (2018).

30 Kim, Y. *et al.* Highly stable contact doping in organic field effect transistors by dopant-blockade method. *Adv. Funct. Mater.* **30**, 2000058 (2020).

31 Xu, Y., Minari, T., Tsukagoshi, K., Chroboczek, J. A. & Ghibaudo, G. Direct evaluation of low-field mobility and access resistance in pentacene field-effect transistors. *J. Appl. Phys.* **107**, 114507 (2010).
